# From light to healing: photobiomodulation therapy in medical disciplines

**DOI:** 10.1186/s12967-025-07466-3

**Published:** 2025-12-29

**Authors:** Pooja Shivappa, Shaik Basha, Shimul Biswas, Vijendra Prabhu, Smitha S. Prabhu, Aparna Ramakrishna Pai, Krishna Kishore Mahato

**Affiliations:** 1 Department of Biophysics, Manipal School of Life Sciences, Manipal Academy of Higher Education, Manipal, Karnataka 576104 India; 2 Photoceutics and Regeneration Laboratory, Centre for Microfluidics, Biomarkers, Photoceutics and Sensors (μBioPS), Department of Biotechnology, Manipal Institute of Technology, Manipal Academy of Higher Education, Manipal, Karnataka 576104 India; 3 Department of Dermatology, Venereology and Leprosy, Kasturba Medical College-Manipal, Manipal Academy of Higher Education, Manipal, Karnataka 576104 India; 4 Department of Neurology, Kasturba Medical College-Manipal, Manipal Academy of Higher Education, Manipal, Karnataka 576104 India

**Keywords:** Photobiomodulation therapy (PBMT), Low-level light therapy, Light-emitting diode (LED), Laser therapy, Wound healing, Neuroprotection, Pain management

## Abstract

**Background:**

Photobiomodulation therapy (PBMT) represents a rapidly expanding area of translational research that bridges photophysics, mitochondrial biology, and clinical rehabilitation. It leverages low-level light to modulate cellular bioenergetics, inflammatory signaling, and tissue repair processes across various medical disciplines.

**Methods:**

A systematic literature search was conducted using Google Scholar, Scopus, PubMed, and Web of Science to identify recent clinical and mechanistic studies on PBMT. The gathered evidence was analyzed to evaluate the influence of PBMT on cytochrome c oxidase–mediated energy transduction, reactive oxygen species modulation, nitric oxide signaling, and cytokine regulation.

**Results:**

Current findings indicate that PBMT exerts multifaceted effects on metabolism and immune homeostasis. Clinical applications have expanded from dermatology and wound healing to musculoskeletal, neurological, ophthalmic, and oncologic conditions. PBMT shows potential for symptom alleviation, accelerated recovery, and tissue protection under oxidative or inflammatory stress. However, translation from preclinical evidence to consistent clinical outcomes remains constrained by non-standardized dosimetry, inconsistent energy delivery, and heterogeneous study endpoints. Negative or equivocal outcomes in trials involving trained or low-stress cohorts highlight the context-dependent efficacy of PBMT. Immunoregulatory insights further reveal links between redox-sensitive transcriptional control and systemic cytokine balance.

**Conclusions:**

By integrating mechanistic and clinical perspectives, this review positions PBMT as a promising yet incompletely optimized platform for mechanism-guided phototherapy. Future directions include biomarker-guided treatment monitoring, advanced device engineering, and personalized PBMT modeling through optical and metabolic profiling to achieve its full translational and therapeutic potential.

## Graphical Abstract

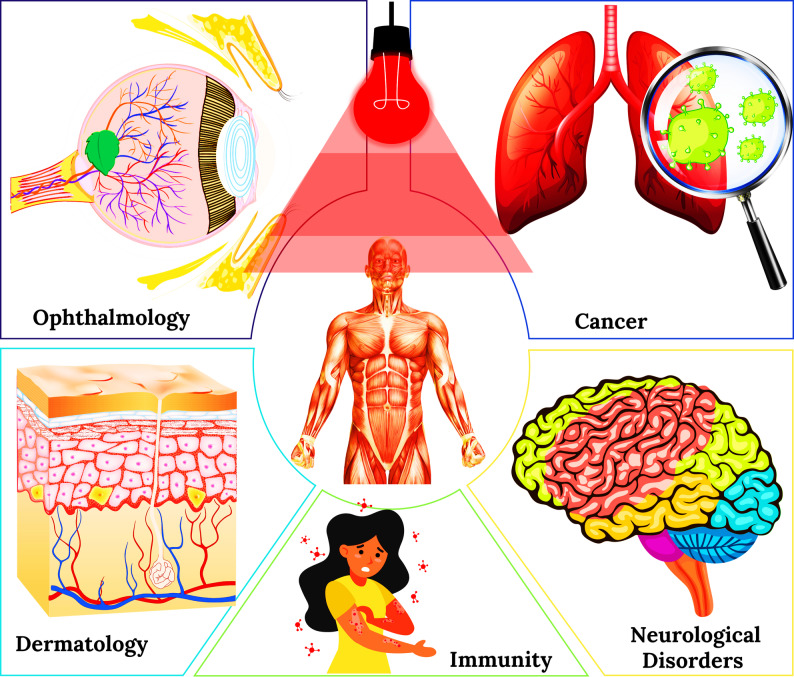

## Background

Over the past five decades, photobiomodulation (PBM), formerly termed low-level laser (light) therapy, has evolved from Mester’s 1967 observation of laser-induced hair regrowth into a versatile, evidence-supported therapeutic modality across multiple medical disciplines [1]. PBM utilizes non-ionizing red to near-infrared (NIR) light, typically within the 600–1100 nm “optical window,” to activate endogenous photoacceptors such as mitochondrial cytochrome c oxidase (CCO) and, in some cases, light-gated ion channels. At the molecular level, photon absorption by CCO initiates electron transport within mitochondria, enhancing adenosine triphosphate (ATP) production and controlling reactive oxygen species (ROS) generation. These bioenergetic changes alter redox balance, release bound nitric oxide (NO), and increase mitochondrial membrane potential and oxygen consumption [2]. The transient ROS burst acts as a secondary messenger, activating key transcriptional pathways (e.g., NF-κB, CREB, and MAPK/ERK) that regulate energy metabolism, angiogenesis, anti-inflammatory responses, and cellular proliferation [3]. PBM also modulates intracellular calcium via transient receptor potential (TRP) channels and mitochondrial calcium fluxes [2], coupling mitochondrial activity to cytosolic signaling through the cAMP and PKC pathways. Collectively, these cascades link photon absorption to systemic bioresponses such as angiogenesis, immunomodulation [4] and neuroprotection [5].

The biphasic dose response, or Arndt–Schulz law, observed across diverse cell types, underscores the importance of optimizing parameters such as wavelength, fluence, irradiance, and exposure time for each tissue and pathology [2, 6]. Early PBM studies were confined to narrow domains such as dermatology and dentistry, but advances in light-emitting-diode arrays, helmet-based transcranial systems, and NIR-II lasers have broadened applications across neurology, oncology, musculoskeletal medicine, cardiovascular therapy, and regenerative modulation. Mechanistically, PBM also engages in mitochondrial retrograde signaling and communication from mitochondria to the nucleus, which influences gene expression related to oxidative stress adaptation and calcium homeostasis [7, 8]. This cross-talk explains how localized mitochondrial effects can translate into tissue-wide and systemic therapeutic responses.

Recent studies have revealed that the effects of PBM extend beyond mitochondrial activation to include vascular and immunomodulatory mechanisms. Targeted light exposure stimulates angiogenesis through the upregulation of vascular endothelial growth factor (VEGF) and hypoxia-inducible factor-1α (HIF-1α); it also suppresses pro-inflammatory cytokines such as tumor necrosis factor-α (TNF-α), interleukin-1β (IL-1β), and interleukin-6 (IL-6), as well as matrix metalloproteinases (MMP-1, MMP-13) that drive cartilage degradation [9, 10]. PBM promotes macrophage polarization from the M1 (pro-inflammatory) phenotype to the M2 (reparative) phenotype. In cardiovascular contexts, particularly within the NIR-II (1,000–1,700 nm) range, PBM enhances endothelial NO bioavailability, improving vasodilation and tissue perfusion [6]. Dermatological meta-analyses provide Level IA evidence for PBM efficacy in radiation dermatitis, oral mucositis, alopecia, and chronic ulcers, which is mediated by the activation of local repair cascades and immune cell modulation [11].

In neurology, both transcranial and intranasal PBM demonstrate neuroprotective effects in Alzheimer’s and Parkinson’s disease models through mitochondrial modulation, enhanced synaptogenesis, and reduced neuroinflammation [7, 8]. Photoactivation of CCO within the 620–760 nm range induces controlled ROS bursts essential for neuronal differentiation and neurite extension; inhibition of CCO or ROS scavenging abolishes these effects [3], directly linking mitochondrial phototransduction to neuronal remodeling. Combination strategies integrate PBM with rehabilitative exercise, neurotrophic factors, or stem-cell therapy, yield synergistic outcomes, underscoring their translational relevance [12]. Despite strong mechanistic and clinical evidence, standardization remains a major challenge. Heterogeneity in dosimetric reporting, incomplete documentation of beam geometry, and underpowered or non-blinded designs limit the reproducibility of results across dermatological, neurological, musculoskeletal, dental, and cardiovascular studies. Discipline-specific reviews, such as those focused on dermatology [11, 13], orthodontics [10], arthritis [9], or neurodegeneration [7, 8], often overlook shared mechanistic features, including conserved mitochondrial targets and biphasic response patterns. Furthermore, regulatory and reimbursement frameworks remain constrained by the absence of internationally harmonized PBM standards, particularly for consumer-grade devices whose commercial expansion outpaces translational validation.

The present review provides a cross-disciplinary synthesis of PBM by integrating mechanistic biology, clinical evidence, and translational challenges across dermatology, age-related complications, such as Alzheimer’s and Parkinson’s disease, which are the the most common diseases in the field of neurology, musculoskeletal, rehabilitation medicine, oncology, supportive care, dentistry, ophthalmology, autoimmune diseases, etc. By embedding recent insights on photon, receptor interactions, mitochondrial signaling, ATP and ROS homeostasis, and calcium-dependent pathways, this background establishes a continuous rationale linking molecular phototransduction to clinical outcomes. Through systematic comparison of mechanisms and treatment parameters across specialties, and by highlighting protocol optimization and deep-tissue targeting, this review advances a unified evidence framework for PBM. Collectively, these insights aim to equip researchers and clinicians with an interdisciplinary reference that aligns mechanistic and clinical progress, supporting PBM’s evolution from an adjunctive to a mainstream, evidence-based therapeutic modality.

## Recent clinical trials and mechanistic studies

Photobiomodulation therapy (PBM) has transitioned from an empirical adjunctive therapy to a scientifically grounded biomedical intervention influencing mitochondrial bioenergetics, neuroplasticity, inflammation, and tissue regeneration. Across diverse organ systems, a consistent mechanistic core has emerged, light-driven activation of cytochrome c oxidase and related mitochondrial pathways, which propagates through neural, vascular, and immune networks. The following synthesis integrates recent mechanistic and clinical investigations, illustrating how foundational mitochondrial mechanisms translate into systemic physiological and clinical benefits across neurological, vascular, and regenerative domains.

The mechanistic essence of PBM lies in its interaction with mitochondrial chromophores, initiating downstream effects that ripple across cellular networks. Modena et al. provided critical clinical evidence of this principle in a study of obese women undergoing red and infrared LED-based PBM prior to bariatric surgery. Using a split-abdomen design and immunohistochemical analysis, they demonstrated robust upregulation of the mitochondrial markers, FAS, FIS1, DRP1, MFN2, OPA1, and cAMP, in treated adipose tissue (*p* < 0.001). These findings not only confirm that PBM stimulates mitochondrial fission–fusion dynamics but also show that photonic stimulation enhances metabolic turnover at the cellular level [14]. This mitochondrial activation forms the biochemical foundation for the systemic effects of PBM.

Supporting this, Wang et al. dissociated photothermal from photochemical effects by comparing EEG responses in participants receiving 1064-nm prefrontal tPBM or equivalent thermal stimulation. PBM selectively enhanced cortical alpha and beta power, signatures of neural excitability and attentional engagement, while heat produced the opposite effect. Together, Modena and Wang established that PBM’s effects are not thermal artifacts but arise from genuine mitochondrial photoactivation that propagates to neural circuitry [15]. These foundational studies provide the bioenergetic logic for the broader therapeutic potential of PBM: if mitochondrial activation augments cellular metabolism, it can equally enhance synaptic signaling, vascular tone, and tissue regeneration. This mechanistic thread is clearly traceable through the following clinical investigations.

Building on these mechanistic insights, clinical studies have explored whether light-induced mitochondrial activation translates into neurocognitive improvement. De Oliveira et al. conducted a randomized, placebo-controlled trial in 93 older adults with mild cognitive impairment (MCI), a condition characterized by synaptic loss and declining neuroplasticity. After 60 days of transcranial near-infrared PBM, participants showed significant cognitive improvement on the MoCA scale (*p* = 0.03) and elevated serum BDNF levels (*p* = 0.0046), with benefits persisting at the 3-month follow-up. No adverse changes were noted in neurodegeneration markers (NSE, S100B) [16]. This study provides clinical validation for PBM-induced mitochondrial–neurotrophic coupling: the same metabolic activation observed by [14]. may enhance neuronal survival and synaptic potentiation through upregulated BDNF signaling.

Similarly, Tang and Jiang revealed how neuromodulatory efficacy of PBM depends not only on wavelength but also on temporal structure. Their randomized, sham-controlled experiment in 56 healthy adults compared 660-nm and 850-nm continuous versus pulsed stimulation at 40–100 Hz. Only the 40 Hz pulsed tPBM produced significant gains in vigilance and working-memory tasks, alongside increased gamma-band EEG power, an electrophysiological signature of cortical synchrony [17]. Together, these studies connect molecular activation with functional neural reorganization: mitochondrial stimulation enhances bioenergetic readiness, which manifests as improved neuroplasticity and cognitive performance at the systems level.

If PBM can upregulate BDNF and synchronize cortical oscillations, it may also influence affective circuits. Noda et al. extended this logic in a double-blind, placebo-controlled crossover trial in patients with mild major depressive disorder (MDD). Participants using 40-Hz violet-light goggles experienced significant reductions in depressive symptoms (MADRS *p* = 0.042) without adverse events [18]. These findings complement the 40 Hz entrainment effects reported by [17], suggesting that rhythmic photic stimulation at gamma frequencies may restore oscillatory balance within limbic–cortical networks. Mechanistically, PBM’s mitochondrial activation likely improves neuronal energy metabolism in prefrontal and thalamic circuits, while its photoneural entrainment component re-establishes synchronous firing patterns associated with mood regulation. Thus, across MCI and MDD, PBM demonstrates a continuum of neurotherapeutic potential, from rescuing synaptic efficiency (BDNF upregulation) to stabilizing network oscillations (gamma coherence). These studies collectively advance PBM as a bioenergetic neuromodulator capable of restoring functional brain dynamics in early neurodegenerative and mood disorders.

The same mitochondrial and neuroplastic mechanisms are evident in sensory systems. Choi and Chang employed a dual-phase preclinical–clinical design to test 830-nm PBM for tinnitus. In mice, PBM reversed sodium-salicylate-induced glutamatergic hyperactivity in the dorsal cochlear nucleus by downregulating VGLUT2, while in humans, trans-tympanic PBM improved tinnitus perception and psychological well-being, although the between-group differences were not statistically significant. This cross-species alignment reinforces PBM’s ability to normalize excitatory neurotransmission; the same cellular stability that supports cognitive and affective recovery may also dampen maladaptive auditory plasticity. The modest clinical effect size suggests that heterogeneity in light penetration and patient pathology still constrain efficacy, but the mechanistic coherence remains compelling [19].

Like cortical neurons, retinal neurons, are energy-hungry cells that are vulnerable to mitochondrial decline. Nassisi et al. explored PBM’s ability to restore retinal metabolism in early and intermediate age-related macular degeneration (AMD). Over six months, single-eye PBM treatment improved visual acuity and contrast sensitivity for up to three months before effects waned, indicating partial reversibility once stimulation ceased [20]. In contrast, Burton et al., using the multi-wavelength LumiThera Valeda^®^ system in the multicenter LIGHTSITE II trial, demonstrated sustained functional gains (+ 4 ETDRS letters, *p* = 0.02) and reduced drusen progression over nine months. The difference likely stems from Burton’s use of multiple wavelengths (590/660/850 nm) and repeated treatment cycles parameters that sustain mitochondrial respiration and delay photoreceptor degeneration [21]. Together, these studies reveal a unifying pattern: PBM enhances performance by restoring mitochondrial efficiency across the brain and retina. Where [16] and [18] targeted synaptic and mood circuits [21] and [20], demonstrated similar rejuvenation in visual pathways, extending PBM’s bioenergetic paradigm from the neural to the sensory domains.

Beyond neural tissues, PBM’s mitochondrial effects translate into improved musculoskeletal and vascular physiology. Navarro-Ledesma et al. conducted a triple-blinded, placebo-controlled trial in patients with fibromyalgia, a condition marked by mitochondrial dysfunction and central sensitization. Twelve sessions of whole-body PBM produced large, durable reductions in pain (*p* < 0.001), improved self-efficacy, and reduced catastrophizing through the six-month follow-up. These long-term effects mirror those seen in cognitive trials, suggesting that PBM’s systemic mitochondrial enhancement attenuates both nociceptive and affective dysregulation [22]. At the tissue level, Murakami-Malaquias-Silva et al. applied an 808-nm PBM during orthodontic molar uprighting, and reported significantly faster tooth movement and modulation of the levels of IL-1β, a key bone-remodeling cytokine [23]. While focused locally, these findings parallel [22] systemic results: PBM appears to accelerate cellular turnover through cytokine-mitochondrial interplay.

In vascular physiology, Hauck et al. assessed endothelial responsiveness in healthy volunteers using bilateral 810-nm PBM over the radial and ulnar arteries. Although intra-group flow-mediated dilation improved (*p* < 0.001), between-group effects were small (*p* = 0.702). These subtle results reinforce the principle that PBM’s circulatory benefits manifest most clearly under pathological or hypoxic stress [24], consistent with [25] findings in diabetic vasculopathy. At the intersection of metabolism and healing, PBM regulates angiogenesis and immune stability. Torkaman et al. conducted a double-blind RCT on 30 diabetic patients with grade II foot ulcers using Ga–As laser PBM (904 nm). PBM accelerated wound closure (%DWSA *p* = 0.003) and normalized angiogenic markers, reducing VEGF levels (*p* = 0.005) while enhancing nitric oxide levels (*p* < 0.001). The inverse correlation between VEGF and the healing rate suggests that PBM restores vascular efficiency, reducing the compensatory VEGF overexpression typical in chronic hypoxia [25].

Similarly, Sediva et al. demonstrated immune stabilization in maxillofacial surgery patients receiving 808-nm PBM: treated individuals maintained postoperative sIgA and lysozyme levels, whereas controls showed significant declines. Combined with computed tomography (CBCT) imaging showing reduced edema, these data confirm the dual role of PBM in vascular regulation and immune equilibrium [26]. The mechanistic continuity from [25] to [26] is clear; both illustrate PBM’s capacity to normalize local inflammation through mitochondrial and redox homeostasis, supporting the same cellular resilience seen in neural and retinal tissues. To translate these cellular benefits into large-scale clinical recovery, Ye and Xiang evaluated 830-nm LED PBM in 145 patients post-blepharoplasty. Compared to traditional care, PBM markedly reduced swelling, pain, and anxiety (all *P* < 0.05) without side effects [27]. This large, randomized trial demonstrated that the molecular and immunological modulations described by [26] can be effectively scaled to cosmetic surgery, yielding tangible patient-centred outcomes. Furthermore, the hemodynamic modulation described by [24] likely contributes to accelerated healing: improved microcirculation expedites edema resolution and oxygen delivery, linking microvascular adaptation to visible recovery.

The translational maturity of PBM is best exemplified in oncology. Ferrari et al. conducted a multicenter observational study in 118 head-and-neck cancer patients receiving radiotherapy or chemoradiotherapy, and reported that daily PBM reduced the overall incidence of oral mucositis (65.2%) and severe grades 3–4 (28%). Logistic regression identified PBM as protective even after adjusting for HPV status and concurrent chemotherapy [28]. Magee et al. implemented a standardized nurse-led PBM program for pediatric patients undergoing hematopoietic stem cell transplantation (HSCT) (*n* = 62 PBM; *n* = 81 controls). PBM significantly reduced mucositis duration and total parenteral nutrition dependence (*p* < 0.001), while lowering supportive-care costs [29]. These pragmatic studies close the mechanistic–clinical loop: the mitochondrial stabilization and anti-inflammatory effects described by [14, 25], and [26] manifest here as reduced mucosal cytotoxicity and faster tissue repair. Crucially [28] and [29], demonstrated PBM’s clinical scalability and safety, transitioning it from controlled experiments to sustainable hospital practice.

Across all studies, PBM consistently acts through a unified mechanistic pathway: mitochondrial photoactivation enhances ATP synthesis and redox signaling [14]. Neuroplastic and neuroimmune modulation yields cognitive and mood improvements [16]. Neurotransmission stabilization mitigates sensory hyperactivity (Choi) and retinal degeneration [20]. Systemic cytokine and vascular regulation underlie pain relief and tissue remodeling [23]. Angiogenic and immunologic normalization accelerates wound repair and recovery [25]. Clinical implementation frameworks [28] validate the reproducibility, safety, and cost-effectiveness of PBM. This evidence collectively traces a clear biological continuum: PBM re-energizes the mitochondrion, re-balances the cell, and re-stabilizes the system, whether neural, vascular, or epithelial.

The methodological rigor has also matured. Early descriptive reports [26] have evolved into multi-center RCTs [21]; [16]; [22], reflecting increasing clinical confidence. Yet, heterogeneity in dosimetry, wavelength combinations, and follow-up duration still limits meta-analytic synthesis, emphasizing the need for standardized PBM reporting guidelines. The cumulative body of evidence demonstrates that PBM’s therapeutic reach is unified by a single mechanistic substrate: mitochondrial bioactivation leading to systemic functional restoration. From cellular respiration [14] to cognitive resilience [16], mood stabilization [18], sensory recovery [21], and tissue regeneration [25], each domain reinforces the role of the PBM as a bioenergetic interface between light and life. Future investigations should pursue mechanistically anchored, long-term randomized trials with standardized dosimetry and cross-domain biomarkers (e.g., BDNF, VEGF, and IL-1β). Integration of imaging, spectroscopy, and metabolic profiling will further clarify dose–response relationships and optimize clinical algorithms.

## Clinical applications in different medical fields

Photobiomodulation therapy (PBMT) has advanced from experimental observations to broad clinical testing across diverse medical fields. By modulating mitochondrial bioenergetics, redox signaling, inflammatory cascades, and tissue remodeling pathways, PBMT offers a non-invasive strategy to influence both local and systemic physiology. Recent studies have investigated its therapeutic potential in conditions ranging from chronic wounds and dermatological disorders to musculoskeletal pain, neurological dysfunction, ocular disease, oncology supportive care, and immune modulation. However, clinical outcomes remain variable, reflecting differences in disease biology, patient selection, and irradiation protocols. The following subsections highlight and critically analyzed PBMT applications across major clinical domains, such as dermatology and wound healing, musculoskeletal and pain management, neurological and cognitive disorders, ophthalmology, oncology, supportive care, and infectious or immune-related conditions, highlighting mechanistic insights, therapeutic benefits, and areas requiring further investigation.

## Dermatology

Photobiomodulation therapy (PBMT), historically termed low‑level laser therapy, has progressed from experimental use in tissue repair to rigorously controlled clinical and translational research. By activating CCO and photoreceptive chromophores within the mitochondrial respiratory chain, PBMT increases adenosine triphosphate formation, moderates reactive oxygen species generation, and orchestrates anti‑inflammatory, analgesic, and regenerative signalling [2, 6]. Across oncology supportive care, reconstructive dermatology, and inflammatory airway disease, investigators have deployed red to near‑infrared (NIR) wavelengths between approximately 630 and 940 nm using lasers or light‑emitting diodes (LEDs) in pulsed or continuous mode. The following investigations span animal, human, and mixed clinical settings, collectively charting PBMT’s methodologic parameters, biological outcomes, and translational traction.

Robijns et al. conducted a multicentre, randomized, placebo‑controlled trial evaluating PBMT for the prevention of severe acute radiation dermatitis (ARD) in 46 head‑and‑neck‑cancer patients during intensity‑modulated radiotherapy. The treatment employed a class IV MLS M6 laser (ASA srl, Italy) with dual laser diodes at 808 nm and 905 nm, average radiant power 3.3 W, and fluence 4 J cm⁻². Each session irradiated a 3.14 cm² field at 0.168 W cm⁻² for 300–600 s, delivered bi‑weekly from the first to last radiotherapy day (14 sessions). Patients wore eye protection; sham treatments used identical protocols without light emission. The PBMT group exhibited a 49% reduction in grade 2–3 dermatitis at the end of treatment, demonstrating significant mitigation of radiodermatitis severity (*p* = 0.002) relative to standard skin care. The quality‑of‑life indices trended higher, although the difference was not statistically significant. The findings established parameter reproducibility, near‑infrared dual wavelengths with moderate fluence in bi‑weekly administration, as clinically effective prophylaxis for radiotherapy‑induced skin toxicity [30].

In accordance with the human data, Park and colleagues assessed PBMT prophylaxis against radiodermatitis in BALB/c mice, and compared 633 nm (visible red) and 830 nm (NIR) light under constant energy density 60 J cm⁻². The mice received 36 Gy X‑irradiation (12 Gy/day for 3 days) and were subsequently treated with HEALITE II LED arrays (50–80 mW cm⁻², continuous wave) every other day. Histopathologic, immunochemical, and apoptotic markers were quantified at 7 and 21 days post‑irradiation. Both wavelengths suppressed epidermal hyperplasia, dermal hypertrophy, and leukocytic infiltration while reducing apoptosis and preserving collagen organization. No differential efficacy between wavelengths emerged, implying that tissue penetration at both red and NIR bands suffices for anti‑inflammatory photobiologic activity [31].

Bensadoun et al. translated the PBM to an easily deployed CareMin650 flexible‑fiber LED system for head‑and‑neck and breast‑cancer cohorts. Prophylactic sessions delivered 650 nm red light at irradiance ≈ 20–28 mW cm⁻², adjusted to 3 J cm⁻²; curative applications used 6 J cm⁻², corresponding to session lengths of 1.5–3 min for oral pads and 2–5 min for dermal pads. Treatments were performed three to five times weekly throughout the course of radiotherapy. Across 1312 exposures in 72 patients, the procedure was devoid of device‑related adverse events. Grade 3 mucositis or dermatitis rarely occurred (≤ 5%), and over 70% of pre‑existing lesions improved or stabilized under therapy. Patient acceptability and operator reproducibility were uniformly high, validating 650 nm LED emission at 3–6 J cm⁻² as a safe clinical standard for mucosal and cutaneous protection in radiotherapy [32].

In aesthetic-dermatology practice, Elawar and colleagues examined a hybrid PBMT–static‑magnetic‑field (PBMT‑SMF) modality in 27 patients presenting with erythema, edema, or pain after pulsed‑dye‑laser or fractional‑radiofrequency facial therapy. The Milta Derm emitter integrated multiple spectral bands (625, 528, 470, 850, 905 nm) with peak power densities ≈ 15–180 mW cm⁻² and magnetic induction 0.5–1 mT. Ten‑minute “anti‑inflammatory” sessions employed red + infrared pulsed light at 1 kHz; “anti‑edema” protocols sequentially combined violet‑IR (1 kHz) and yellow‑IR (15–24 Hz) emissions. Postprocedurally, pain scores declined by an average of 40–47%, erythema typically resolved within 24 h, and edema dissipated within 48 h, markedly shorter than usual recovery. Even though the study was non‑randomized, it illustrates photonic‑magnetic synergy and shorter‑duration irradiations capable of promoting postoperative dermal homeostasis without pharmacologic adjuncts [33].

Recently, a double‑blind, randomized trial investigated the effects of local and transcutaneous (modified ILIB) PBMT on inflammatory manifestations of cellulite in 25 women. Ten 650 nm LED pads (100 mW, 0.5 cm² emitter area) were used to deliver 6 J cm⁻² for 5 min to each hip region, thrice weekly over four weeks; concurrent systemic illumination targeted the radial arteries bilaterally at the same wavelength for 30 min (180 J total). Combined local + ILIB therapy elevated pressure‑algometric pain thresholds by ≈ 32%, exceeded the 8–20% increase from local PBMT alone, and reduced the mean skin surface temperature by 1.2 °C versus 0.4 °C (thermal imaging). These data underscore a dose‑response and field‑size dependency, where prolonged systemic irradiation potentiates local anti‑inflammatory temperature normalization and analgesia [34].

Another randomized, placebo‑controlled double‑blind trial enrolled 62 patients with persistent allergic rhinitis. The active group received dual‑wavelength intranasal PBMT at 660 nm + 808 nm, 6 J per nostril, plus 1 J infrared 808 nm at seven external nasal points (total 7 J/session). Each treatment lasted ~ 70 s and was applied twice weekly for four weeks. Photometric and questionnaire evaluations revealed significant physiological gains: peak nasal inspiratory flow improved (*p* < 0.001), nasal‑obstruction scores decreased (*p* = 0.048), and rhinitis control indices increased (*p* = 0.035). Olfactory performance (UPSIT) remained unchanged, suggesting that intranasal light absorption primarily moderates mucosal inflammation rather than sensory neurons. The 660–808 nm combination effectively amalgamated superficial and deeper penetration within tolerable fluence levels [35].

Complementary to the above findings, Lang‑Illievich and co‑workers tested PBMT’s antipruritic potential in a double‑blind, split‑body human model using 640 nm red light (Repuls 7, 175 mW cm⁻², 6‑minute continuous exposure) on histamine‑ and Mucuna pruriens‑evoked itch zones. In 17 healthy volunteers, each serving as self‑control, PBMT yielded pronounced reductions in histamine‑mediated itch intensity (Δ 13.9 VAS points, *p* < 0.001), contraction of the flare area (Δ 0.18 cm²), and attenuation of alloknesis (–0.8 cm, *p* < 0.001). Thermal readings revealed negligible changes, confirming non‑thermal action. In contrast, Mucuna‑induced (non‑histaminergic) itching was unaffected, delineating a mechanistic boundary wherein PBMT interrupts histamine‑dependent neuro‑inflammatory pathways but not protease‑activated, C‑fiber‑selective pruritus [36].

Across these investigations, the irradiation parameters spanned visible red (625–670 nm) to near‑infrared (808–905 nm) spectra, irradiances of 15–175 mW cm⁻², and dose densities of 3–60 J cm⁻², which were delivered by continuous or low‑frequency pulsed modalities. Despite the heterogeneity of indications, such as radiodermatitis, mucositis, postoperative inflammation, chronic rhinitis, pruritus, and subcutaneous panniculopathy, the convergent tissue responses are strikingly consistent: attenuation of the inflammatory infiltrate, vascular normalization, pain or itch relief, and accelerated restitution of cutaneous and mucosal integrity. Clinical parameters such as frequency (2–5 sessions weekly for 4–7 weeks) and fluence per field ≈ 3–6 J cm⁻² for the epithelium or 30–60 J cm⁻² for deep tissue have emerged as reproducible benchmarks. Collectively, the data corroborate the position of PBMT as a versatile, safe, light‑dose‑dependent therapy that is optimally tuned within the red–NIR window to control inflammation and restore tissue function without thermal injury.

Despite consistent evidence of the therapeutic efficacy of PBMT across diverse biological contexts, significant challenges persist that temper its seamless clinical adoption. Among these obstacles is the absence of standardized dosimetric guidelines; variations in wavelength, fluence, irradiance, exposure time, and pulse characteristics often result in inconsistent therapeutic outcomes across laboratories and devices. Tissue optical complexity further complicates reproducibility, as penetration depth and scattering differ between epithelial, muscular, and vascular tissues, limiting precise energy delivery to deep targets. Biological heterogeneity, particularly in human trials compared with animal or in-vitro studies, introduces further variability in systemic responses to PBMT. Mechanistically, the interplay between mitochondrial photoreception, reactive oxygen species generation, and transcriptional modulation remains incompletely defined, giving rise to both under- and overstimulation risks. Additionally, concerns persist regarding the use of PBMT in oncological contexts, where parameter-dependent outcomes range from increased tumor apoptosis to undesirable proliferation, underscoring the need for wavelength- and dose-specific safety validation. Moving forward, the field must prioritize multi‑center calibration efforts, real‑time dosimetry standardization, and integrative bio‑optical modeling to reconcile cellular photochemistry with clinical biophysics. Combining PBMT with adjunctive modalities such as pharmacological sensitizers, static magnetic fields, or systemic laser blood irradiation represents a promising frontier, contingent upon rigorous translational frameworks and precision control of photon-tissue interactions.

## Wound healing

**Fig. 1:**
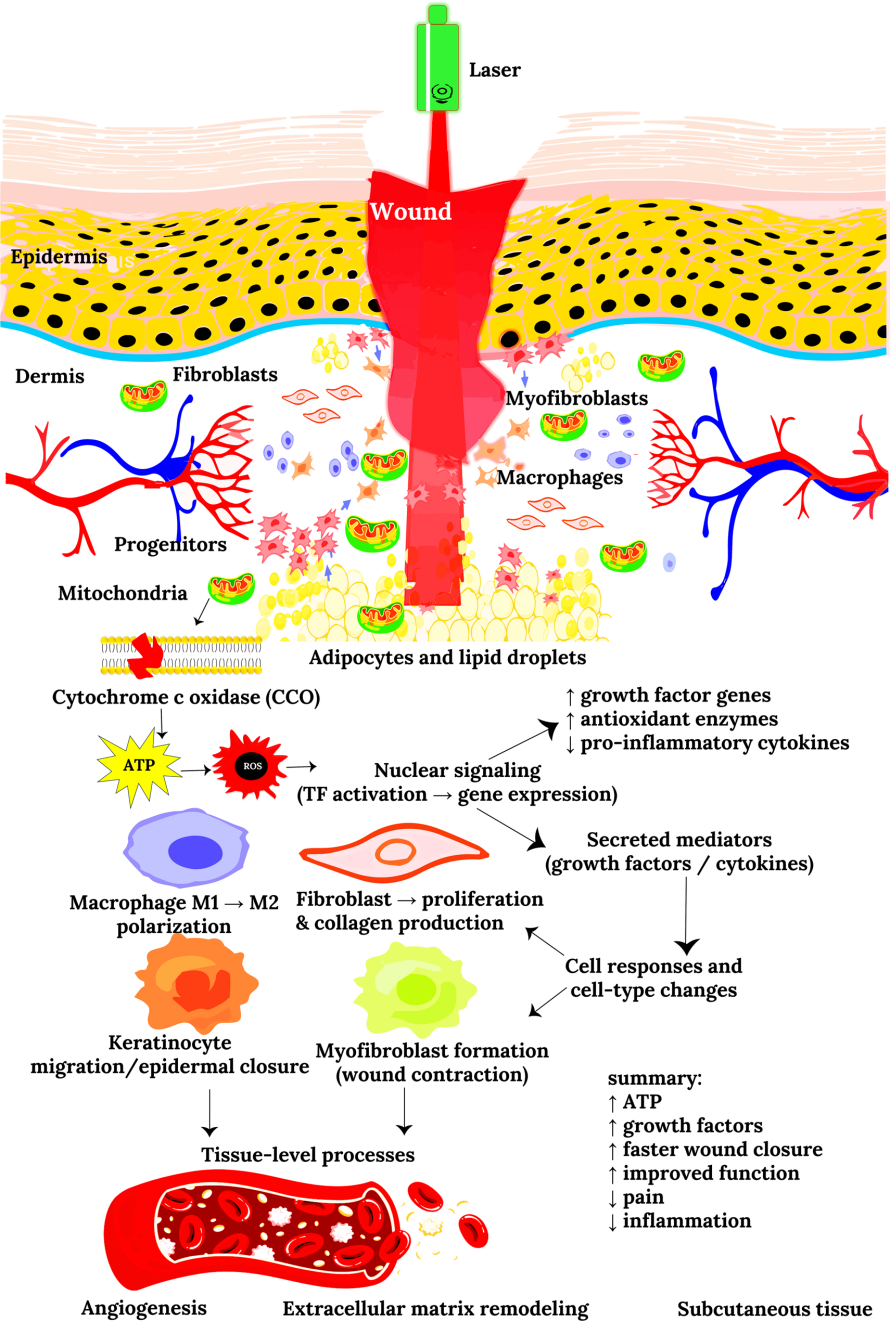
Photobiomodulation (PBM)–mediated wound healing at the molecular, cellular, and tissue levels. PBM (600–1100 nm) penetrates the epidermis, dermis, and subcutaneous tissue, where light is absorbed by mitochondrial chromophores such as cytochrome c oxidase (CCO), leading to enhanced ATP synthesis and controlled reactive oxygen species (ROS) signaling. These changes activate transcription factors (NF-κB, AP-1, and HIF-1α), driving the expression of growth factors (TGF-β, VEGF, and IGF-1), antioxidant enzymes, and anti-inflammatory cytokines while suppressing pro-inflammatory mediators (TNF-α and IL-1β). The resulting cellular responses include fibroblast and keratinocyte proliferation, macrophage polarization toward a reparative phenotype, myofibroblast activation, collagen synthesis, and extracellular matrix remodeling. At the tissue level, these mechanisms promote angiogenesis, reduce edema and oxidative stress, accelerate wound closure, and enhance overall healing outcomes

Photobiomodulation (PBM) represents an evolving interdisciplinary paradigm bridging photophysics and tissue biology to expedite cellular regeneration, manage inflammation, and restore tissue homeostasis. When examined across multiple experimental modalities, from infra‑blue to near‑infrared laser systems and low‑power LED arrays, PBM demonstrates both consistency and adaptability in orchestrating the molecular architecture of wound repair. The studies highlighted in this section, although diverse in model systems and dosimetry, collectively define a narrative of convergent bioenergetic enhancement, fibroblast activity modulation, and collagen matrix remodeling across tissue types ranging from oral and dermal mucosa to diabetic ulcers and stem cell cultures. The mechanism of PBM is graphically represented in Fig. 1.

At the translational interface, Soliman and colleagues employed a multiwavelength LED configuration (465 nm blue, 640 nm red, 880 nm near‑infrared) following fractional ablative resurfacing in human skin, illustrating the synergistic potential of simultaneous chromatic stimulation. Operating at an irradiance of 6.5 mW cm⁻² and a fluence of 11.7 J cm⁻² for thirty minutes thrice weekly, the treatment aimed to exploit the modulatory influence of blue light on nitric oxide dynamics, red‑light‑driven fibroblast proliferation, and near‑infrared‑mediated mitochondrial excitation through cytochrome c oxidase. While objective metrics such as erythema reduction and epithelial restitution trended toward improvement, statistical confirmation remains limited by dosage and cohort size. Nonetheless, the work established the feasibility of a multiplexed spectral regimen and underscored the central hypothesis that multiband photonic exposure may harmonize superficial antimicrobial effects with deep‑tissue metabolic activation [37].

Comparative LED analysis by Zhao et al. advanced this mechanistic understanding by comparing the effects of 630 nm and 810 nm LED-mediated PBM on wound healing both in *vitro* and in vivo. The results demonstrated that irradiation with both 630 nm and 810 nm LEDs significantly enhanced the proliferation of mouse fibroblast (L929) cells across different irradiance levels (1, 5, and 10 mW/cm²). The proliferation rate increased with increasing exposure duration (100, 200, and 500 s) but declined when irradiation exceeded 500 s, indicating a biphasic dose–response effect. Furthermore, both the 630 nm and 810 nm LED treatments at 5 mW/cm² markedly improved the fibroblast migration capacity, with no significant difference observed between the two wavelengths. In vivo experiments revealed that both 630 nm and 810 nm LED irradiations promoted faster wound closure and upregulated the expression of vascular endothelial growth factor (VEGF) and transforming growth factor (TGF) in the wounded skin of type 2 diabetic mice. Collectively, these findings suggest that LED-mediated PBM facilitates diabetic wound healing by enhancing fibroblast proliferation, migration, and the expression of key growth factors, with both 630 nm and 810 nm LEDs demonstrating comparable therapeutic efficacy [38].

By continuing the mechanistic thread linking mitochondrial activation to transcriptional reprogramming, Kim et al. demonstrated that 808 nm laser exposure (63.69 mW cm⁻² for 240 s per session, five sessions totaling ≈ 60 J) in rat skin wounds reduced lesion size within three to seven days while stimulating collagen accumulation and neoepithelial thickening. Central to these findings was the correlation between phosphorylated cyclic‑AMP response‑element binding (CREB) protein and the TGF‑β/TNF‑α ratio, suggesting that CREB phosphorylation functions as a nodal mediator that translates mitochondrial photon absorption into nuclear transcriptional control. The alignment between the p‑CREB/CREB ratio and histological progression reinforced PBM’s capacity to synchronize inflammatory resolution with the proliferative phase of repair, reflecting a chain from oxidative phosphorylation enhancement through CREB‑p300/CBP co‑activation to collagen gene transcription [39].

The regenerative and paracrine implications of these mitochondrial changes were described by Mirzaei Seresht et al., who investigated the effects of PBM at two different wavelengths, 650 nm and 810 nm, on human adipose-derived stem cells (ADSCs) and their secreted exosomes under in vitro conditions. To assess the therapeutic outcomes, cell viability, proliferation, and exosome characteristics were evaluated using MTT assays, scanning electron microscopy (SEM), bicinchoninic acid (BCA) assays for exosomal protein quantification, and Dynamic Light Scattering (DLS) for determining exosome size distribution. The findings revealed that PBM at both 650 nm and 810 nm wavelengths significantly enhanced ADSC viability and proliferation, as demonstrated by MTT assay results (*p* = 0.008 for 650 nm and *p* = 0.007 for 810 nm). Moreover, exosomal analysis revealed that PBM-treated groups produced exosomes with higher protein concentrations and an average particle size of approximately 86 nm, indicating improved exosome quality. These findings highlight the potential of PBM in augmenting ADSC-mediated tissue repair and chronic wound healing, although further mechanistic and clinical studies are warranted to elucidate the underlying molecular pathways and optimize treatment parameters for translational applications [40].

The findings concerning the oral regenerative axis are consistent with those of Thakur et al., who examined healthy and diabetic gingival fibroblasts by assessing cell proliferation, migration, and gene expression profiles. PBM was performed using a 940 nm diode laser at 100 mW power for 20–40 s through a 300 μm fiber tip. The cellular responses were assessed through assays evaluating proliferation, viability, and migration, along with the expression of key genes. The results demonstrated that cell viability and proliferation were higher in the fibroblast groups exposed to three PBM sessions of 20 s each, wheaeas the smallest in vitro wound gap at 24 h post-treatment was observed in the 20-second exposure group. Gene expression analysis revealed that in healthy gingival fibroblasts, PBM for 40 s increased FGF2, TGFα, and FOXO1 expression by approximately 30-fold, 13-fold, and 9-fold, respectively, whereas in diabetic gingival fibroblasts, TGFα expression was elevated by 11-fold and 13-fold following 20- and 40-second exposures, respectively. Although these differences were not statistically significant, the overall findings suggest that three PBM sessions using a 940 nm diode laser at 100 mW for 20 s may enhance cellular proliferation, migration, and regenerative potential. Further studies are warranted to optimize the dosimetry parameters to maximize the therapeutic efficacy of PBM at this wavelength [41].

The oral model, intertwined with a precedent set by Astuti et al., investigated the effects of red (649 nm, 4 J/cm²) and blue (403 nm, 8 J/cm²) diode laser treatments on post-extraction wound healing in rats through histopathological and immunohistochemical analyses. Histopathological analysis revealed that red diode laser treatment significantly increased the number of lymphocytes and fibroblasts and increased angiogenesis compared with those in the control groups. Immunohistochemical findings demonstrated a marked upregulation of Collagen-1α, indicating that stimulated collagen synthesis is essential for new tissue formation, along with a reduction in IL-1β expression, suggesting reduced inflammation. Although blue laser treatment also has beneficial effects on wound healing, its lower degree of tissue penetration limits its efficacy compared with that of red laser treatment. Overall, the results indicate that red diode laser irradiation at 649 nm effectively accelerates the proliferative phase of wound healing following molar extraction, as evidenced by enhanced cellular activity, collagen formation, and angiogenesis [42].

Extending the red spectrum to a pure LED context, Schmidt et al. investigated the effects of red-spectrum LED therapy on normal skin keratinocytes (HaCaT cells) and full-thickness dorsal wound healing in Wistar rats to elucidate its therapeutic mechanisms. An LED device in a plate format measuring 6 × 3 × 0.6 cm, composed of six light emission points with a wavelength of 660 ± 20 nm and a power of 5 mW, was used for treatment. Each irradiation session lasted 7 min, delivering an energy density of 2.7 J/cm², equivalent to 2 J per point. In vitro, HaCaT cell viability and migration were assessed using SRB and scratch assays under stress (2.5% FBS) and optimal (10% FBS) conditions, and LED-treated and sham-irradiated groups were compared. In vivo, fifty rats with induced full-thickness dorsal wounds were divided into LED-treated and sham groups and received daily irradiation. Tissue samples collected on days 3, 5, 10, 14, and 21 were analyzed for oxidative stress (MDA, SOD, GSH) and cytokines (IL-1β, IL-10, and TNF-α). LED therapy significantly enhanced HaCaT cell viability, promoted faster inflammation resolution, reduced MDA levels, increased the GSH content, and decreased IL-1β, IL-10, and TNF-α expression by day 10, although re-epithelization remained unaffected. Overall, LED irradiation at 660 ± 20 nm effectively accelerated wound healing by modulating inflammation and enhancing antioxidant defense, demonstrating its potential as a safe and non-invasive therapeutic approach for skin repair [43].

Despite collective evidence positioning photobiomodulation as a highly promising biophysical therapy for wound repair, several methodological and translational challenges continue to constrain its universal clinical adoption. One of the foremost difficulties lies in the variability of dosimetric parameters across studies; differences in wavelength bandwidth, exposure duration, output power, and delivery geometry can produce heterogeneous bioeffects ranging from stimulatory to inhibitory outcomes. The biphasic dose dependence observed in fibroblast and stem cell models emphasizes the criticality of energy density optimization; however, at present, there is no consensus framework for defining the therapeutic window across different tissue depths and pathological states. Furthermore, the mechanistic heterogeneity revealed by various model systems, from CREB‑mediated transcriptional responses in rat wounds to exosome modulation in cultured stem cells, underscores a lack of standardized biological endpoints for benchmarking PBM efficacy. Translationally, while near‑infrared light demonstrates deep tissue penetration with robust mitochondrial activation, its clinical reproducibility remains hindered by variations in device calibration, patient skin phototypes, and thermal accumulation during repeated exposures.

The field must advance toward integrative standardization and mechanistically grounded personalization. Future research should couple high‑precision dosimetry with bio‑optical modeling of photon transport to tailor the wavelength and fluence to targeted chromophore layers within specific wound environments. Multi‑omics profiling and live‑cell imaging could delineate the temporal kinetics of ROS, NO, and cytokine modulations, establishing dynamic biomarkers of PBM responsiveness. Clinical translation will further benefit from hybrid LEDs and laser systems that combine spectral flexibility with real‑time tissue feedback to prevent photoinhibition and optimize energy delivery. Importantly, large‑scale, multicentric trials using harmonized irradiation parameters and robust histological and biochemical endpoints are indispensable to bridge the gap between bench mechanistic clarity and bedside therapeutic precision. Through such concerted efforts, PBM can evolve from an empirically guided adjunct into a predictable, quantifiable, and customizable modality for regenerating diabetic, oral, and cutaneous wounds.

## Musculoskeletal management

Photobiomodulation (PBM) therapy has emerged as an increasingly recognized non-invasive, non-pharmacologic modality for managing a wide array of musculoskeletal and pain-related conditions. Its biological foundation lies in the ability of near-infrared and red-light photons to penetrate biological tissues and interact with chromophores, particularly CCO in the mitochondrial respiratory chain. This interaction promotes electron transfer, enhances ATP synthesis, and modulates reactive oxygen species (ROS), and nitric oxide (NO) signaling, ultimately influencing downstream transcriptional events that govern cell proliferation, inflammation control, and pain modulation. These cellular and molecular mechanisms collectively support tissue repair, neuromodulation, and the restoration of homeostasis following injury or chronic inflammation [2, 6].

**Fig. 2:**
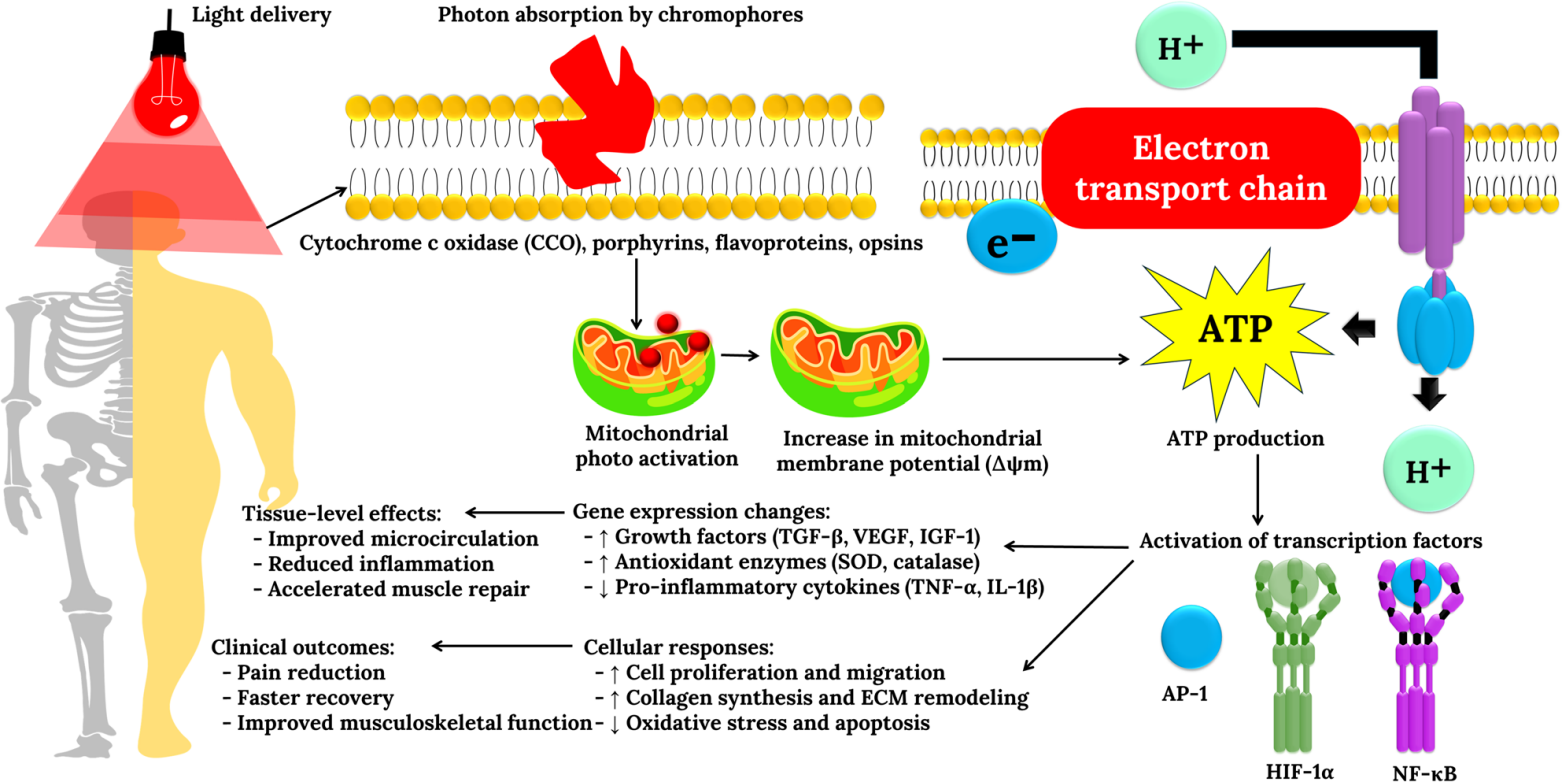
Molecular mechanism of photobiomodulation (PBM) therapy in musculoskeletal management. PBM uses red to near-infrared light (600–1100 nm) absorbed by mitochondrial chromophores such as cytochrome c oxidase (CCO), enhancing electron transport chain activity, ATP production, and controlled ROS signaling. These events activate transcription factors (NF-κB, AP-1, and HIF-1α), leading to the upregulation of growth factors and antioxidant enzymes, the downregulation of pro-inflammatory cytokines, and the promotion of cell proliferation, collagen synthesis, and extracellular matrix remodeling. At the tissue level, these responses improve microcirculation, reduce inflammation, and accelerate repair, resulting in pain reduction and enhanced musculoskeletal function

Over the past decade, many randomized controlled trials (RCTs) have explored PBM in terms of postoperative recovery, degenerative joint diseases, fibromyalgia, temporomandibular disorders (TMDs), exercise-induced muscle damage, and neuropathic pain. Although most studies demonstrate significant therapeutic benefit, others report inconsistent or null outcomes, underscoring the complexity of translating photobiological principles into clinical efficacy. These discrepancies reflect heterogeneity in dosimetry parameters (wavelength, energy density, frequency, duration, and site of application), variability in patient characteristics, and differences in concurrent interventions such as exercise and physical therapy and their molecular interplay is graphically represented in Fig. 2.

The perioperative domain has provided some of the most compelling clinical evidence for the role of PBM in modulating inflammation and facilitating soft tissue recovery. Hadad et al. conducted a randomized, double-blind, split-mouth clinical trial involving 13 patients who underwent bilateral third molar extraction. Using an 810 nm diode laser delivering 6 J (100 mW, 60 s per point) intraorally at four sites, they reported significantly lower pain scores on the PBM-treated side than did sham irradiation at 24 h (7.56 ± 6.25 vs. 32.25 ± 22.78; *p* < 0.001) and 48 h (19.47 ± 9.27 vs. 39.87 ± 4.21; *p* = 0.011). Moreover, edema reduction was notable at 24 and 48 h, indicating effective modulation of acute inflammatory edema. The strength of the study lies in its controlled bilateral design and repeated-measures statistical approach, which minimizes interindividual variation. These findings indicate that PBM therapy effectively reduces postoperative pain and edema following third molar surgery, supporting its role as an adjuvant modality in improving surgical recovery outcomes [44].

Parallel outcomes were reported in major maxillofacial procedures. In a randomized controlled study, D’Avila et al. examined PBM administered with a 940 nm laser post-orthognathic surgery across 20 patients randomized into treatment and control groups. The PBM regimen included immediate postoperative application, sessions at 24 and 48 h, followed by weekly sessions for four weeks. The PBM-treated group exhibited progressive and statistically significant pain reduction within 24 h, complete resolution by week three (*p* < 0.001), and notable improvements in trismus at days 14 and 30 (*p* = 0.002 and *p* = 0.019, respectively). Although the differences in paresthesia were not statistically significant, the direction of the effect favored PBM. These findings extend PBM’s clinical utility from minor dental extractions to major orthognathic reconstructions, demonstrating that wavelength-specific infrared light penetrates deep orofacial tissues, enhancing neural recovery and muscular relaxation through mitochondrial bioactivation [45].

In chronic musculoskeletal pain syndromes, PBM’s ability to modulate central sensitization pathways has been most rigorously tested in fibromyalgia. This complex syndrome, characterized by widespread pain, sleep disturbances, and fatigue, has shown responsiveness to both PBM and its combination with a static magnetic field (PBMT-sMF). A landmark triple-blind, randomized, placebo-controlled clinical trial by Ribeiro et al. at the Laboratory of Phototherapy and Innovative Technologies in Health (LaPIT), Brazil, enrolled 90 female patients(45 PBMT-sMF; 45 placebo). Across nine sessions over three weeks, PBMT-sMF treatment led to a highly significant reduction in tender points and improved clinical impact scores (*p* < 0.0001), with no adverse events. The static magnetic field likely synergized with PBM by modulating ionic fluxes across cell membranes, stabilizing mitochondrial potentials, and promoting sustained NO release, collectively enhancing analgesia and microcirculatory perfusion. This study represents one of the most robust demonstrations of PBM bioenergetic modulation in chronic systemic pain syndromes [46].

Despite robust independent effects, several trials have reported no additive benefit when PBM is combined with exercise therapy. Understanding this apparent lack of synergy is critical for refining multimodal rehabilitation protocols. However, not all studies on fibromyalgia have revealed synergistic outcomes when PBM is combined with conventional physical therapies. Vassão et al. conducted a double-blind RCT involving 51 women with fibromyalgia, who were randomized into four groups: Control (*n* = 12), active PBM (*n* = 12), aerobic exercise + olacebo PBM (*n* = 13), and aerobic exercise + active PBM (*n* = 14). The aerobic exercise protocol comprised supervised ergometric cycling sessions, whereas PBM was delivered via a cluster device using an incremental dosage protocol (20 J, 32 J, and 40 J) twice weekly for 12 weeks. All intervention arms showed significant intragroup improvement in pain reduction and quality of life (*p* < 0.05). However, no significant intergroup differences were detected. These findings suggest that both aerobic exercise and PBM therapy independently contribute to symptom improvement in fibromyalgia patients [47].

The apparent lack of synergy between PBM and exercise therapy may stem from overlapping mechanistic pathways. Both interventions stimulate mitochondrial biogenesis through the upregulation of PGC-1α and NRF-1, increase vascular endothelial growth factor (VEGF) expression, and reduce the expression of inflammatory cytokines such as IL-6 and TNF-α. When concurrently applied, these shared pathways may reach a physiological plateau, resulting in a “ceiling effect,” wherein additional photobiostimulation yields no incremental gains [48]. Moreover, aerobic exercise transiently increases muscle perfusion and oxygenation, potentially reducing photon retention and absorption efficiency during PBM irradiation [49]. Differences in session timing (e.g., concurrent versus sequential application) also critically affect outcomes. When PBM is applied immediately post-exercise, increased tissue temperature and hemodynamics may alter light penetration and photochemical conversion efficiency [50].

To overcome these challenges, optimization strategies should consider staggered PBM-exercise protocols. Pre-exercise PBM could prime cellular energetics, enhance oxygen utilization and delay fatigue onset, whereas post-exercise PBM may better target secondary inflammatory responses and oxidative stress. Additionally, dose-dependent calibration, ensuring that PBM fluence remains within the biphasic dose-response window, is essential to prevent overstimulation of mitochondrial pathways that can paradoxically induce oxidative imbalance. Future clinical protocols should incorporate individualized dosimetry informed by muscle composition, skin pigmentation, and baseline redox status, as these parameters directly influence photon absorption and therapeutic efficacy. The integration of near-infrared spectroscopy (NIRS) to monitor muscle oxygenation in real time could further refine PBM delivery timing relative to exercise-induced metabolic fluxes.

Evidence in osteoarthritis (OA) adds another dimension to understanding PBM’s context-specific efficacy. Jorge et al. conducted a large double-blind, placebo-controlled RCT involving 127 participants with knee OA, randomized into Exercise-only (*n* = 41), exercise + active PBM (*n* = 44), and exercise + placebo PBM (*n* = 42) groups. Treatments were delivered thrice weekly for eight weeks, with follow-ups at immediate, three-month, and six-month intervals. While all groups exhibited significant pain reduction and functional improvement from baseline, no between-group differences were found at any time point, reinforcing the dominant role of structured exercise therapy in OA management. These findings suggest that although exercise training alone effectively alleviates symptoms in knee OA, the addition of PBM does not confer incremental therapeutic benefits, emphasizing the central role of exercise as the primary intervention in managing knee OA-related pain and disability [51].

A contrasting perspective is offered by N. C. Pinto et al., who introduced an innovative personalized PBM dosimetry model in a pilot RCT with 31 patients suffering from chronic knee Osteoarthritis (OA). The individualized approach adjusted thetotal energy output (526–1402 J per session using an 850 nm laser) according to body mass index and skin pigmentation, thereby normalizing the photon absorption efficiency. Treatments were administered twice weekly for five weeks, and the results demonstrated significant and sustained pain relief from the fourth session onward, accompanied by improvements in quality-of-life indices, dopamine levels, and peripheral microcirculation. These findings underscore that personalized PBM dosimetry, tailored to optical and metabolic characteristics, can produce superior therapeutic outcomes even in conditions where generic PBM protocols show limited efficacy [52].

In addition to degenerative and chronic pain conditions, PBM has been increasingly examined as an ergogenic and recovery-enhancing modality in sports and exercise physiology. In an elegant double-blind, placebo-controlled crossover study, Giovanini et al. investigated the preconditioning effects of PBM on repeated-sprint performance in ten elite basketball players. Using dual-wavelength LED irradiation at 660 and 850 nm with an energy density of 12 J·cm⁻² (83.4 J per point across ten points), athletes underwent a standardized warm-up, active or sham PBM, and ten 30-meter sprints with directional changes. The results revealed no significant differences between the PBM and placebo conditions in total sprint time (*p* = 0.662; ES = − 0.06), best sprint time (*p* = 0.869; ES = 0.02), fatigue index (*p* = 0.169; ES = 0.64), or sprint decrement (*p* = 0.124; ES = − 0.75). Similarly, the mean (*p* = 0.687; ES = 0.07) and maximal (*p* = 0.837; ES = − 0.03) heart rates did not differ between conditions. These findings indicate that PBM preconditioning, under the tested parameters, does not enhance repeated-sprint ability or cardiovascular responses in elite basketball players, suggesting the limited ergogenic potential of PBM for high-intensity intermittent performance [53].

Conversely, in a triple-blind, placebo-controlled crossover trial, H. D. Pinto et al. evaluated PBM combined with a static magnetic field (PBMT-sMF) in twelve trained CrossFit^®^ athletes, testing four different temporal protocols (before, after, or both before and after workout). The study’s comprehensive design included biochemical markers such as creatine kinase (CK), interleukin-6 (IL-6), and oxidative stress indices (carbonylated proteins, TBARS), alongside antioxidant enzymes (catalase, superoxide dismutase). The results demonstrated that PBMT-sMF applied either before or after WOD significantly enhanced functional test performance immediately post-exercise and at 24 and 48 h (*p* < 0.05) compared to placebo and the combined before-and-after PBMT-sMF condition. Moreover, PBMT-sMF applied before or after workout of the day (WOD) significantly reduced creatine kinase activity, oxidative stress markers, and inflammatory mediators, while increasing antioxidant enzyme activity (all *p* < 0.05). Compared with the placebo, PBMT-sMF applied both before and after WOD also reduced creatine kinase at 24 h and IL-6 levels at 24 and 48 h. No adverse events were reported. Collectively, these findings indicate that PBMT-sMF, when applied either pre- or post-exercise, effectively enhances recovery, reduces muscle damage and inflammation, and mitigates oxidative stress in CrossFit^®^ athletes, supporting its role as a potent ergogenic and recovery-enhancing intervention in high-intensity functional training [54].

In contrast to findings in trained athletes, F. Ma et al. conducted a randomized controlled trial that investigated the effects of photobiomodulation (PBM), low-intensity stretching, and their combination on delayed-onset muscle soreness (DOMS) and performance in untrained adults. Fifty-four participants were assigned to the control, PBM, stretching, or PBM + stretching groups after eccentric exercise. Interventions were applied 24, 48, and 72 h post-exercise, and the outcomes included the pressure pain threshold (PPT), perceived soreness (NRS), single-leg forward jump (SLFJ), and maximal isometric voluntary contraction (MIVC). Compared with the control group, the PBM + stretching group presented a lower PPT (greater pain sensitivity) at 48 (*p* = 0.037) and 72 h (*p* = 0.045), with no significant differences in the NRS score, MIVC score, or SLFJ score (*p* ≥ 0.052). DOMS did not correlate with strength or performance (*p* ≥ 0.09), indicating that PBM and stretching, alone or combined, did not attenuate soreness or enhance recovery, and that the combination might even heighten pain sensitivity [55].

In addition to sports and rehabilitation, PBM has shown promising outcomes in post-surgical neuropathic recovery. In a randomized clinical study, Chuah et al. examined 105 carpal tunnel release (CTR) patients, 56 of whom were randomized into PBMT or control arms. PBM (808 nm, 9 J per session for 3 min) was administered ten times over three weeks. The PBMT group exhibited greater improvements in Boston Carpal Tunnel Questionnaire scores (*p* = 0.018), morning pain (*p* = 0.019), and pinch strength at multiple follow-up points (*p* < 0.05), along with improved sensory discrimination at three and six months. These findings provide compelling evidence for PBM’s neuromodulatory capacity, likely mediated by enhanced Schwann cell proliferation, axonal transport, and local microcirculation, which translates into accelerated recovery of both sensory and motor function. Importantly, the study’s six-month follow-up confirms the durability of PBM’s neural effects, an underexplored but clinically vital dimension [56].

Similarly, Abufoul et al. conducted a prospective, double-blind RCT evaluating self-applied PBM in 50 patients following rotator cuff arthroscopic surgery (RCAS). With the use of an 808 nm B-Cure Laser Pro device (16.5 J/cm² for 15 min daily over 3 months), the PBM group demonstrated significantly faster pain reduction at three and six months (*p* = 0.040, *p* = 0.038), superior functional recovery (QuickDASH ΔFU-6 M: 30 ± 24 vs. 18 ± 14; *p* = 0.029), and greater quality-of-life improvements. Notably, a higher proportion of PBM patients achieved the Minimal Clinically Important Difference (MCID) and Patient Acceptable Symptom State (PASS), confirming meaningful patient-perceived benefits. These results suggest that low-intensity, continuous home PBM can effectively complement standard physiotherapy, promoting sustained neuroplastic and musculoskeletal healing through cumulative mitochondrial activation and a reduced oxidative load in tendons and periarticular tissues [57].

The efficacy of PBM has also been explored in temporomandibular disorders (TMDs), which are complex conditions involving both muscular and joint dysfunction. In a well-designed RCT of 54 women, Furquim et al. compared PBM applied at palpation-determined pain points versus fixed anatomical landmarks, using a 780 nm laser at 4–6 J once weekly for four weeks. The pain-point application produced significantly greater reductions in pain intensity (*p* = 0.0002) and Short-Form McGill Pain scores (*p* = 0.004), independent of dose. This evidence confirms that precision-targeted PBM, guided by patient-specific nociceptive mapping, optimizes light delivery to the most metabolically active and inflamed regions, enhancing local mitochondrial repair and nociceptor inhibition. It also highlights the need to individualize PBM parameters not only by physical attributes but also by pain localization and tissue pathology [58].

Collectively, these findings demonstrate that PBM’s efficacy in musculoskeletal management is context- and dose-specific. Its benefits are most pronounced when it is applied to tissues exhibiting metabolic stress, oxidative imbalance, or inflammatory dysregulation, conditions in which mitochondrial activation, NO modulation, and ROS normalization confer meaningful functional recovery. Conversely, in physiologically healthy or optimally trained tissue, the incremental benefit of PBM may be limited. Importantly, the heterogeneity across studies reflects the absence of a standardized clinical framework integrating wavelength, fluence, and treatment frequency with condition-specific pathology. The integration of PBM with exercise or electrotherapy shows conditional synergy, with outcomes contingent on precise temporal sequencing and dosimetric optimization.

The evidence strongly supports PBM as a versatile and mechanistically grounded modality for musculoskeletal rehabilitation, which is capable of modulating inflammation, enhancing mitochondrial function, and accelerating recovery across a wide range of conditions. Its clinical effectiveness depends on individualized parameterization, including wavelength, energy density, application site, and timing relative to co-interventions such as exercise. The absence of synergy with exercise in certain trials likely reflects mechanistic redundancy and requires optimized sequencing strategies rather than the abandonment of combination therapy. Future directions should emphasize personalized dosimetry models, biofeedback-guided PBM delivery, and integration with biomarker-based endpoints (e.g., oxidative stress markers, cytokine panels) to enable precise, reproducible, and condition-tailored interventions. This refinement will transform PBM from an empirical adjunct to a mechanistically informed, evidence-driven component of musculoskeletal and pain rehabilitation medicine.

## Neurological and cognitive disorders

**Fig. 3:**
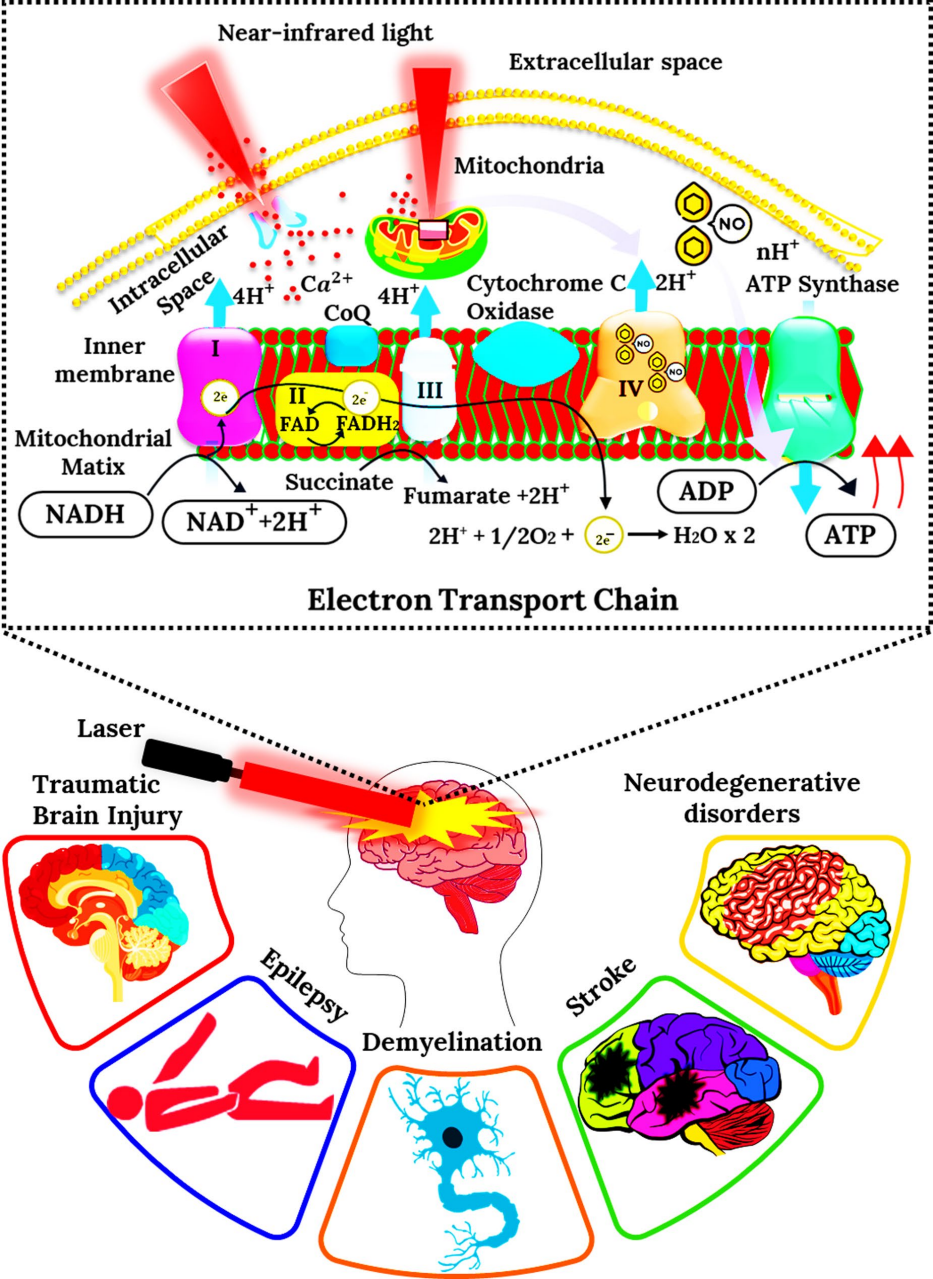
Schematic representation of photobiomodulation (PBM) therapy illustrating its cellular and neurological effects. The upper panel depicts the mechanism by which near-infrared light penetrates cellular membranes to target mitochondria, enhancing mitochondrial electron transport chain activity and ATP synthesis through cytochrome c oxidase stimulation. The lower panel demonstrates the potential therapeutic applications of PBM for various neurological disorders, including traumatic brain injury, neurodegenerative diseases, stroke, epilepsy, and demyelination, highlighting the laser-mediated modulation of brain function

Neurological and cognitive disorders, including Alzheimer’s disease (AD), Parkinson’s disease (PD), Multiple sclerosis (MS), and stroke, collectively represent a leading cause of disability and mortality worldwide [59–61]. Despite significant advances in neuropharmacology, most current therapies are symptomatic rather than restorative. The pursuit of translationally viable, non-invasive, and mitochondria-targeted strategies has therefore intensified. Within this landscape, photobiomodulation (PBM), the controlled use of low-level visible and near-infrared (NIR) light (600–1100 nm), has emerged as a compelling therapeutic modality. The biological rationale of PBM lies in its ability to stimulate mitochondrial cytochrome c oxidase (CCO) and enhance ATP production, redox balance, and calcium signaling while attenuating oxidative stress and neuroinflammation [2]. The mechanism of PBM is graphically represented in Fig. 3.

The photonic energy absorbed by CCO increases the electron transport chain efficiency and promotes nitric oxide (NO) dissociation, improving local oxygen consumption and microcirculation. Collectively, these actions foster cellular resilience, synaptic plasticity, and neurovascular coupling, which are critical for neuronal repair and cognitive preservation [2]. However, despite an expanding body of evidence, the translational relevance of PBM remains hindered by methodological heterogeneity, and differences in wavelength, fluence, and treatment regimen yield variable results across disorders. To address this gap, this integrative review synthesizes and critically analyzes recent mechanistic and clinical studies, delineating how PBM’s bioenergetic, anti-inflammatory, and neurovascular actions converge to produce functional recovery in neurological disease.

At the cellular level, PBM activates mitochondria-dependent transcriptional programs that underlie neuronal survival. Li et al. conducted an elegant transcriptomic investigation in murine models treated with transcranial PBM (808 nm, 1 h × 30 days). Whole-brain RNA-seq identified over 2,400 differentially expressed genes, 1,005 in the hippocampus and 1,482 in the cortex, implicating approximately twenty neurodegeneration-related pathways. The therapy downregulated the expression of oxidative-stress mediators (NF-κBIα, JUN, JUND), inflammatory factors (IL-1RAPL1, TNFαIP6), and apoptotic markers (CASP3, AKT3, CDKN1A). Strikingly, genes central to amyloid precursor protein (APP) processing (BACE1, BACE2, PSEN2, and APH1B) were suppressed, leading to a measurable reduction in the cortical APP concentration. These results provide the first large-scale molecular evidence that PBM mitigates Alzheimer-linked cascades by coupling mitochondrial redox regulation with transcriptomic remodeling [62].

Complementing these intracellular mechanisms, Lin et al. revealed a vascular–lymphatic dimension of PBM neuroprotection. Using a D-galactose ageing mouse model, the authors showed that 1275-nm PBM restored meningeal lymphatic vessel (MLV) integrity, enhancing the brain clearance of advanced glycation end products (AGEs), which are highly prevalent in diseases like diabetes and cardiovascular diseases [63, 64] and reducing oxidative stress. Improved lymphatic drainage was accompanied by elevated nitric-oxide–dependent vasodilation and a notable improvement in learning and memory performance. This work extends PBM’s relevance beyond cellular metabolism, suggesting that it rejuvenates glymphatic and meningeal clearance systems, which are key in neurodegenerative toxin removal [65]. Together, the studies of [62] and [65] form the mechanistic cornerstone of PBM therapy, linking mitochondrial bioenergetics and neurovascular clearance as dual drivers of neural recovery.

The mechanistic rationale for PBM’s neuromodulatory action has catalyzed multiple translational studies in Alzheimer’s continuum disorders. Razzaghi et al. conducted a 12-week pilot clinical study in patients with AD and mild cognitive impairment (MCI). Compared with a slight decline in controls, daily 810-nm PBM sessions (20 min) led to a statistically significant functional improvement in the Disability Assessment for Dementia (Δ = +4.3 ± 4.9; *p* = 0.041), whereas anxiety and depression remained unchanged. These results imply that PBM may enhance functional autonomy before measurable cognitive changes, aligning with early metabolic and neurovascular restoration [66]. In a larger, double-blind study, Chun et al. assessed at-home, self-administered transcranial PBM (infrared) six times weekly for 12 weeks in individuals with MCI due to AD. The treatment produced a significant rise in Korean Montreal Cognitive Assessment (K-MoCA) cognitive scores and trends toward improved Mini-Mental State Examination performance, without adverse events. The safety profile and scalability highlight PBM’s practicality as a home-based intervention. Mechanistically, these improvements likely reflect sustained mitochondrial activation and enhanced cerebral perfusion, as evidenced in animal models [67].

Shorter-term effects were examined by Zhao et al. in subjects with subjective cognitive decline (SCD), a prodromal stage of AD characterized by preclinical synaptic dysfunction. Targeted PBM over the prefrontal cortex for six days improved working-memory accuracy (88.6% vs. 79.6%) and reduced reaction times (544.8 ms vs. 592.9 ms, *p* = 0.003). The sleep quality indices, while not significantly altered between groups, trended toward improvement within the PBM cohort, suggesting the modulation of frontal cortical sleep networks [68]. Taken together, the studies of [66, 67], and [68] illustrate a continuum of translational efficacy, from early metabolic compensation in SCD to measurable cognitive and functional gains in MCI and AD. Critically, the heterogeneity of these outcomes underscores several translational insights. First, dose and wavelength standardization are pivotal; PBM’s penetration depth and photon flux vary exponentially with wavelength. Second, treatment frequency appears to modulate neuroplastic outcomes: longer regimens, such as the [67] 12-week protocol, yield greater sustained effects than brief interventions do. Third, regional targeting, particularly of prefrontal and hippocampal areas, aligns with observed improvements in executive function and memory consolidation. The convergence of molecular data from [62] with these human outcomes reinforces PBM’s mechanistic plausibility in cognitive restoration.

The translational promise of PBM extends beyond Alzheimer’s pathology to neurodegenerative movement disorders, particularly PD, where mitochondrial dysfunction, oxidative stress, and dopaminergic neuronal loss drive progressive motor decline. Mitochondrial impairment within substantia nigra pars compacta neurons parallel the bioenergetic deficits the PBM targets, positioning it as a potential disease-modifying therapy [69]. In a seminal long-term follow-up, Liebert et al. examined eight PD patients who had participated in an earlier PBM pilot study, reassessing them after five years of continued at-home therapy. Seven patients maintained regular transcranial PBM three times per week, while one discontinued after one year. Remarkably, five of the six continuously treated participants demonstrated either improvement or no decline in MDS-UPDRS III motor scores, accompanied by measurable gains in gait speed, stride length, balance, and cognition. No adverse events were recorded. These outcomes deviate significantly from the expected trajectory of PD progression, where an annual motor decline of 2–3 UPDRS points is typical. These data suggest that chronic PBM exposure may stabilize mitochondrial function and synaptic integrity, mitigating disease progression [70].

Further mechanistic and feasibility confirmation came from Herkes et al., who performed a randomized, double-blind, sham-controlled crossover trial using a helmet-based tPBM system that emits red and infrared light (24 min × 6 days/week × 12 weeks). Treatment adherence exceeded 90%, demonstrating excellent patient compliance. Participants receiving active PBM exhibited consistent reductions in MDS-UPDRS III scores (mean 21.3 → 16.5) compared with minimal changes in the sham arm, although between-group significance was limited by sample size. Importantly, no serious adverse events occurred, and minor transient effects (brief weakness, fine-motor slowing) resolved spontaneously [71]. Together, the studies by [70] and [71] provide complementary evidence, one showing durability and translational feasibility, and the other confirming safety and short-term clinical benefit. Their combined data align with preclinical findings that PBM enhances mitochondrial ATP synthesis, dopaminergic neuron viability, and striatal network coherence, supporting a mechanism-based neuroprotective hypothesis.

Peripheral neuropathies, although outside the central nervous system, share mechanistic parallels with CNS injury, ischemic stress, demyelination, and axonal degeneration. Dong Wu et al. investigated whether laser acupuncture combined with PBM could enhance neural recovery in patients with chronic Bell’s palsy (>8 weeks in duration). In this randomized controlled trial involving 84 patients, Class IV laser treatment (72 sessions) produced significant improvements in House–Brackmann grading (OR = 0.11, *p* < 0.001) and in electrophysiologic markers such as electroneurography (ENoG) and electromyography (EMG) across multiple facial muscles. Enhanced Sunnybrook scores and blink reflex normalization further substantiated motor recovery [72]. These outcomes point toward PBM’s capacity to accelerate remyelination and axonal regeneration through mitochondrial reactivation and upregulated growth-factor signaling. From a translational viewpoint [72], highlighted the applicability of PBM in peripheral nerve repair, complementing its central neuroprotective effects. The convergence of mechanisms, ATP elevation, reactive-oxygen-species moderation, and vascular improvement illustrates a unifying mitochondrial paradigm underpinning both peripheral and central neural restoration.

Stroke recovery remains a key target for PBM translation, as conventional rehabilitation often yields incomplete functional restitution. The multimodal effects of photobiomodulation, which enhance mitochondrial bioenergetics, neurovascular coupling, and cortical reorganization, make it an ideal adjunctive therapy for post-stroke neuroplasticity. Paolillo et al. combined transcranial PBM with neuromuscular electrical stimulation (NMES) in hemiplegic stroke patients to test synergistic rehabilitation potential. The PBM protocol utilized multi-wavelength clusters (660, 808, 980 nm; 648 J per session) applied to 15 head regions. When paired with NMES-induced limb movement, participants exhibited significant improvements in cognitive function, dexterity, pain reduction, and psychosocial well-being, all of which contribute to enhanced quality of life. Ex vivo optical analyses confirmed adequate intracranial light penetration, confirming its mechanistic feasibility. The synergy observed likely reflects metabolic priming of cortical neurons by PBM, which then respond more robustly to NMES-driven synaptic activation, a translational model for combined photonic and electrical rehabilitation [73].

Complementing these findings, Marcele Florêncio das Neves et al. performed a double-blind clinical trial evaluating 780-nm PBM on the paretic upper limb of post-stroke patients with spastic hemiparesis. Following the ten sessions, subjects exhibited a 38% reduction in pain, decreased blood lactate levels, and a 46% increase in elbow-extension range of motion (ROM). These physiological gains indicate enhanced muscle oxidative metabolism and fatigue resistance, which is consistent with increased mitochondrial activity and improved microcirculation. Interestingly, electromyographic activity did not change significantly, suggesting that the benefit of PBM arises primarily from metabolic rather than electrophysiologic modulation of muscle performance [74]. Further insight into systemic vascular effects was offered by Ming-Wei Lai et al., who retrospectively analyzed 34 ischemic-stroke patients receiving intravascular laser irradiation of blood (ILIB) at 632.8 nm alongside standard rehabilitation. The ILIB group demonstrated greater improvement in modified Rankin Scale (mRS) scores (*p* = 0.028) and consistent, though non-significant, gains in functional mobility indices (Barthel Index, 6-minute walk test, Fugl-Meyer Assessment). Notably, no adverse events were reported, underscoring the safety of PBM in vascular-compromised populations [75].

Taken together, the studies of [73, 74], and [75] show PBM’s evolving translational niche in neurorehabilitation. Mechanistically, PBM likely acts through three converging pathways: Bioenergetic activation, which restores mitochondrial ATP and reduces lactate accumulation; neurovascular facilitation, which enhances regional blood flow and angiogenesis; and Neuroplastic reinforcement, increasing cortical excitability and synaptic remodeling when combined with physical or electrical therapy. These collective results validate the promise of PBM as a metabolic and vascular amplifier within multidisciplinary stroke rehabilitation paradigms. However, heterogeneity in wavelength, dose, and treatment scheduling remains a major obstacle to protocol reproducibility. Synthesizing across PD, Bell’s palsy, and stroke cohorts reveals a coherent translational pattern: PBM consistently improves motor performance, coordination, and muscle endurance, with effect magnitude correlating with treatment duration and light dosage. The mechanism is not disorder-specific but reflects mitochondrial and microcirculatory normalization, which enhances neural firing stability and tissue oxygenation.

Comparatively, chronic neurodegeneration (PD) benefits from sustained, low-intensity PBM that preserves neuronal integrity over years [70] and [71], whereas acute or subacute conditions (stroke, Bell’s palsy) respond to short-term, higher-fluence PBM that accelerates regenerative cascades. This duality underscores PBM’s bi-phasic dose response; sub-therapeutic energy fails to trigger photoacceptor activation, while excessive energy may induce oxidative load. Hence, translational optimization requires precise calibration of the fluence and exposure frequency tailored to the disease stage and tissue depth. From a broader systems perspective, these findings suggest that PBM operates as a metabolic neuromodulator, harmonizing neuronal, glial, and vascular interactions across the neuroaxis. This unified mechanism positions PBM as a candidate for cross-indication therapeutic development, warranting rigorous mechanistic imaging and biomarker studies to confirm pathway convergence.

Demyelinating diseases such as MS represent a crucial frontier for PBM translation, as mitochondrial failure, oxidative stress, and chronic neuroinflammation are key mediators of axonal degeneration and fatigue. The potential of PBM to restore cellular energy metabolism while suppressing inflammatory cytokine production provides a compelling biological rationale for its use in MS-associated motor and metabolic dysfunction [76]. Mitra Rouhani et al. conducted a rigorous two-part randomized, double-blind, crossover trial exploring the dose-dependent effects of PBM on skeletal muscle performance in people with multiple sclerosis (pwMS). Using irradiation at 600–1100 nm, the investigators demonstrated that a high dose (120 J) significantly improved tibialis anterior muscle force recovery (101.9% ± 13.55% vs. 96.3% ± 18.48% placebo; *p* = 0.03), while fatigue indices remained unchanged. In the second phase, two weeks of individualized PBM dosing further increased the maximal voluntary contraction strength (185.56 ± 33.95 N vs. baseline 162.7 ± 37.5 N; *p* = 0.01). These results provide quantitative evidence that PBM enhances muscle energetics and contractile efficiency, likely through the restoration of the mitochondrial membrane potential and redox equilibrium within fatigued muscle fibers. Importantly, these improvements were achieved without adverse effects, underscoring the safety and translational viability of PBM as an adjunct therapy for MS-related weakness [77].

However, efficacy may depend critically on treatment parameters and site specificity. Tamiris Silva et al. performed a pilot randomized trial applying sublingual and radial-artery PBM (808 nm, 36 J, 360 s) to address fatigue in relapsing–remitting MS. Contrary to Rouhani’s findings, no significant changes were observed in the Modified Fatigue Impact Scale (MFIS) scores (*p* >0.05). These results suggest that systemic PBM routes may fail to deliver sufficient photon energy to target neuromuscular mitochondria or central fatigue circuits, highlighting the importance of localized irradiation. The discrepancy between Rouhani and Silva emphasizes that the therapeutic efficacy of PBM depends on tissue-specific dosimetry and photon penetration, parameters that are often underreported yet essential for reproducibility [78].

Ageing-related neurological decline involves cumulative mitochondrial damage, vascular dysfunction, and impaired waste clearance, all of which PBM has been shown to modulate. In preclinical ageing models, Lin et al. previously demonstrated that 1275-nm PBM rejuvenated meningeal lymphatic drainage and attenuated oxidative stress, improving cognitive outcomes [65]. By extending this paradigm to sensory systems, Enrico Borrelli et al.explored PBM’s effect on dry age-related macular degeneration (dAMD), a leading cause of vision loss in elderly individuals. Using the EYE-LIGHT^®^ dual-wavelength system (590 and 630 nm), Borrelli et al. randomized 76 patients with early or intermediate dAMD to receive active or sham PBM for four weeks. Treated eyes exhibited a 20.3% rate of ≥ 5-letter visual acuity improvement versus 8.9% in controls (*p* = 0.043), alongside a significant reduction in drusen volume (*p* = 0.013) and excellent tolerability (only mild transient ocular irritation in 20% of cases). These clinical outcomes mirror PBM’s mechanistic benefits observed in neural tissues, enhanced mitochondrial respiration, improved oxygen utilization, and reduced reactive oxygen species burden. The parallel between cortical and retinal responses suggests a shared photonic bioenergetic mechanism across neural systems [79].

At the molecular level, PBM consistently enhances mitochondrial respiration via cytochrome c oxidase activation, increasing ATP generation and stabilizing the membrane potential. The resulting upregulation of redox-sensitive transcription factors (e.g., CREB and NF-κB) leads to secondary transcriptional cascades that suppress apoptosis and inflammation [62]. Concurrently, nitric oxide release from CCO augments local vasodilation, improving perfusion and tissue oxygenation. At the tissue level, PBM amplifies neurovascular and lymphatic homeostasis. Improved microcirculation enhances nutrient delivery and metabolic waste clearance, while activation of meningeal lymphatics [65] reduces neurotoxin accumulation. This dual action fosters an environment conducive to synaptic repair and neuroplasticity. At the systemic and functional level, these cellular and vascular benefits translate into improved cognitive performance, motor recovery, and functional independence across disorders. Clinical data demonstrate enhanced memory and executive function in cognitive impairment [67]; [66]; [68], preserved mobility in PD patients [70], accelerated neurorehabilitation in stroke patients [73], improved neuromuscular recovery in MS [77], and restored sensory function in ageing [79]. The convergence of outcomes across these diverse pathologies reinforces PBM’s role as a systemic bioenergetic modulator rather than a disease-specific intervention.

Despite compelling biological plausibility and early clinical success, PBM research remains constrained by methodological variability. Across the reviewed studies, only a minority employed double-blind randomization [70, 71], and sample sizes were often limited (< 50 participants). Treatment parameters, particularly wavelength, fluence, and session frequency, vary widely, complicating meta-analytic interpretation. Moreover, reporting of study design elements such as control illumination, energy density, and anatomical targeting was inconsistent, echoing concerns about evidence quality. Future translational trials should prioritize the following standardization of dosimetry (wavelength, energy density, exposure time, beam geometry); mechanistic biomarkers, integrating neuroimaging, spectroscopy, and mitochondrial assays to validate target engagement; stratified clinical endpoints that link mechanistic and behavioral improvements, thereby bridging basic and clinical neuroscience.

Across preclinical and clinical evidence, photobiomodulation has emerged as a powerful modulator of neural bioenergetics, inflammation, and vascular function. The reviewed studies collectively demonstrate that PBM enhances cognitive function, motor recovery, and sensory performance through convergent mechanisms involving mitochondrial activation, neuroimmune regulation, and neurovascular rejuvenation. In line with the translational ethos of Translational Medicine, PBM represents a paradigm shift from symptom management to systems-level restoration. The integrated insights from [62] through [79] define a coherent mechanistic–clinical axis that spans ageing, neurodegeneration, and neurorehabilitation. As optimization and standardization advance, PBM has the potential to transform the therapeutic landscape of neurological medicine, bridging light-based biophysics with molecular neuroscience to achieve functional recovery and neuroprotection across the lifespan.

## Ophthalmology

**Fig. 4:**
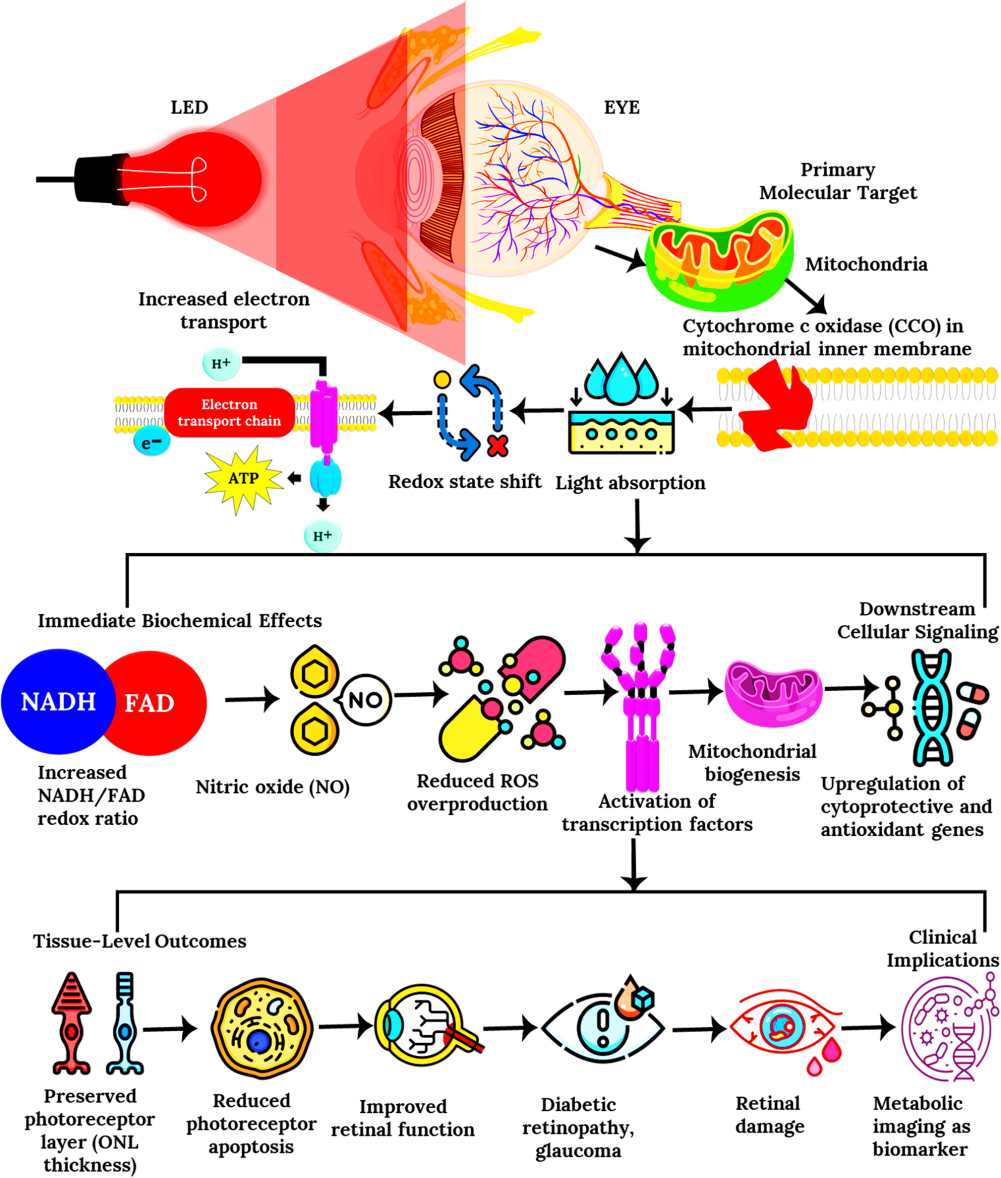
Mechanisms and retinal effects of photobiomodulation (PBM). Near-infrared (830 nm) PBM targets cytochrome c oxidase in retinal mitochondria, improving electron transport, increasing the NADH/FAD redox ratio, and enhancing ATP production while reducing oxidative stress. These bioenergetic changes activate cytoprotective transcription factors, upregulate antioxidant pathways, and preserve photoreceptor structure and function, as shown by a thicker outer nuclear layer (ONL) and improved ERG responses in the P23H retinitis pigmentosa model

The emerging application of repeated low-level red light (RLRL) and multiwavelength photobiomodulation (PBM) therapy has opened a new frontier in controlling ocular growth and retinal degenerative processes. Across multiple randomized clinical trials (RCTs) and mechanistic investigations, converging evidence indicates that targeted red to near-infrared light exposure (590–850 nm) exerts a bioenergetic effect on the retina and choroid through mitochondrial activation, enhanced choroidal perfusion, and modulation of retinal metabolic signaling, collectively translating into measurable structural and functional benefits. The following synthesis integrates findings from studies in a mechanistic continuum, distinguishing their methodological designs, irradiation parameters, and outcomes. The mechanism of the PBM is graphically represented in Fig. 4.

The LIGHTSITE III multicenter RCT evaluated the LumiThera Valeda Light Delivery System, which delivered multiwavelength PBM at 590, 660, and 850 nm to both macular regions over nine sessions every four months, with each session lasting several minutes. One hundred subjects (148 eyes) with nonexudative age-related macular degeneration (AMD) were randomized (2:1) to receive PBM or sham therapy and followed for 13 months. PBM-treated eyes demonstrated a significant improvement in Best-Corrected Visual Acuity (BCVA) (+ 5.4 letters vs. +3.0 letters in controls, *P* = 0.02) and a 9.4-fold reduction in new-onset geographic atrophy. The therapy was well-tolerated with no phototoxicity or inflammation. Mechanistically, PBM activated CCO within the mitochondrial respiratory chain, enhancing ATP production and oxidative phosphorylation efficiency. This photochemical effect stabilized retinal pigment epithelium (RPE) metabolism, promoted nitric oxide (NO)–mediated vasodilation, and restored choriocapillaris perfusion, collectively slowing photoreceptor apoptosis. This trial provides compelling Class I evidence linking mitochondrial photoactivation to preserved retinal function and structure [80].

In one of the first multicenter pediatric myopia RCTs, Jiang et al. examined 264 children aged 8–13 years randomized to RLRL therapy or control (single-vision spectacles). The RLRL device emits 650 nm red light at 0.29 mW retinal power and ~ 1600 lx luminance, is classified as Class I ocular-safe, and is administered for 3 min twice daily (≥ 4 h apart, 5 days/week) for 12 months. Compared with the control group, the RLRL group exhibited axial elongation of 0.13 mm (Δ = 0.26 mm, *P* < 0.001), and myopic progression of − 0.20 D vs. −0.79 D. OCT imaging confirmed that there was no retinal damage. The authors proposed that light penetrated to the RPE and outer retina, stimulating mitochondrial CCO and ATP synthase, and enhancing metabolic efficiency and oxygen utilization. This improvement in retinal-choroidal perfusion reduced hypoxia-induced scleral remodeling, thereby inhibiting ocular elongation. The study’s multicenter, single-blind design and large sample size confer high evidentiary strength for mitochondrial bioenergetics as a therapeutic axis in refractive control [81].

Furthermore, Zhou et al. conducted a 12-month RCT (*n* = 50; ages 8–12 years) in which low-level PBM (650 nm, 6 min/day) was tested against spectacle correction. After one year, axial length decreased slightly (− 0.02 ± 0.11 mm) in the PBM group versus + 0.48 ± 0.16 mm in the control group (*P* < 0.001). SER improved by + 1.25 D relative to baseline. Although subfoveal choroidal thickness (SFChT) did not differ significantly, these structural changes suggest that PBM induced metabolic stabilization within photoreceptors and RPE mitochondria that was sufficient to alter ocular growth kinetics. This study reinforces the reversibility of axial elongation via noninvasive optical bioactivation, a concept previously considered implausible. The authors hypothesized that cytochrome oxidase–driven ROS modulation and mitochondrial biogenesis enhance retinal resilience, supporting perfusion and scleral stiffness without pharmacologic intervention [82].

In a premyopia prevention RCT (*n* = 278; grades 1–4; age 6–11 years), RLRL was introduced in children with SERs between − 0.50 D and + 0.50 D, at risk for myopia. The participants received 650 nm light for 3 min twice daily, 5 days/week for 12 months. The incidence of myopia was 40.8% in the RLRL group vs. 61.3% in the control group, representing a 33–54% relative risk reduction depending on compliance. Axial elongation (0.30 mm vs. 0.47 mm) and SER shift (− 0.35 D vs. −0.76 D) significantly decreased with treatment. OCT analyses confirmed that there was no structural or phototoxic damage. Mechanistically, the authors proposed that RLRL increases retinal and choroidal blood flow, ameliorating scleral hypoxia, a known driver of axial elongation. The enhanced mitochondrial respiration and oxygen diffusion likely stabilize ocular growth signals even before structural myopia develops. This preventive evidence suggests that RLRL engages early metabolic homeostasis to maintain ocular integrity during the premyopic stage [83].

Using swept-source OCT imaging, Xiong et al. conducted a secondary analysis (*n* = 120) from the multicenter RLRL trial (ClinicalTrials.gov NCT04073238) to evaluate macular choroidal thickness (mCT) as a biomarker of therapeutic efficacy. The children in the intervention group received RLRL therapy at 650 nm for 3 min per session, with 2 sessions per day, and a minimum interval of 4 h between sessions. The RLRL-treated eyes demonstrated sustained choroidal thickening (+ 14.7 μm at 1 month, stabilizing to + 9.1 μm at 12 months), whereas the controls showed progressive thinning (− 10.4 μm). Importantly, mCT thickening at 3 months predicted 12-month myopia control (AUC 0.71–0.78), independent of baseline refraction. These findings suggest that PBM-induced mitochondrial activation elevates NO bioavailability and enhances choroidal perfusion, thereby improving oxygen and nutrient flux to scleral tissues. This vascular-metabolic coupling may explain the ability of therapy to reduce axial elongation. This trial not only validated choroidal thickness as a predictive biomarker but also revealed that microvascular perfusion modulation is a mechanistic intermediary between mitochondrial signaling and macroscopic refractive outcomes [84].

In line with previous studies, a single-center, single-masked RCT (*n* = 62) directly compared RLRL therapy (650 nm, 3 min × 2/day) with 0.01% atropine eye drops over 12 months. Axial elongation was markedly lower in the RLRL group (0.08 mm vs. 0.33 mm, *P* < 0.001), as was myopia progression (− 0.03 D vs. −0.60 D). While atropine modulates muscarinic receptor–mediated ciliary signaling, RLRL acts through non-visual phototransduction pathways within retinal mitochondria, restoring bioenergetic balance without affecting accommodation or pupil diameter. The authors inferred that light-induced upregulation of mitochondrial complex IV and NO synthase results in enhanced choroidal blood flow, yielding superior myopia control without pharmacologic side effects. This head-to-head comparison highlights distinct mechanistic domains, neuromodulatory versus photobiological, demonstrating that PBM achieves comparable or greater efficacy via intrinsic energy restoration rather than receptor blockade [85].

Extending RLRL therapy to a high-myopia population, Xu et al. enrolled 192 children (6–16 years, SER ≤ − 4.0 D) in a multicenter, randomized, parallel-group clinical trial (NCT05184621). The intervention utilized a 650 nm, 1600 lx device (0.29 mW retinal output) administered for 3 min twice daily, 7 days/week. After 12 months, axial shortening >0.05 mm occurred in 53.3% of the treated eyes, while controls elongated by + 0.34 mm on average. The mean SER change was − 0.11 D (vs. −0.75 D). These effects were achieved without any structural or functional adverse events. The authors postulated that red light activates mitochondrial oxidative phosphorylation within retinal neurons and scleral fibroblasts, normalizing extracellular matrix turnover and reducing biomechanical expansion. These findings demonstrate that even in severe myopia, photo-induced mitochondrial signaling and vascular perfusion synergy can reverse or halt ocular elongation, a milestone in noninvasive high-myopia management [86].

Collectively, these trials delineate a convergent bioenergetic mechanism: (1) Mitochondrial photoactivation, via CCO absorption of 650–850 nm photons, enhances ATP synthesis and reduces oxidative stress. (2) Perfusion enhancement, secondary to NO-mediated vasodilation, improves choroidal oxygenation, addressing scleral hypoxia. (3) Retinal signaling stabilization, including the modulation of photoreceptor-RPE metabolic cross-talk, reduces pro-myopiagenic signaling. These cellular events translate to macroscopic outcomes: reduced axial elongation, stabilized refraction, improved BCVA, and delayed retinal degeneration. Importantly, the integration of mitochondrial and vascular mechanisms clarifies their relative contributions: mitochondrial bioenergetics provide the initiating trigger for energy restoration, while choroidal perfusion acts as the sustaining mediator that maintains tissue oxygenation and biomechanical homeostasis. The cross-study coherence, spanning preventive (premyopia), corrective (low/moderate myopia), and degenerative (AMD) contexts, highlights that photobiomodulation fundamentally rebalances retinal metabolism, underscoring its translational potential as a safe, repeatable, and mechanistically unified optical therapy.

## Oncology supportive care

**Fig. 5:**
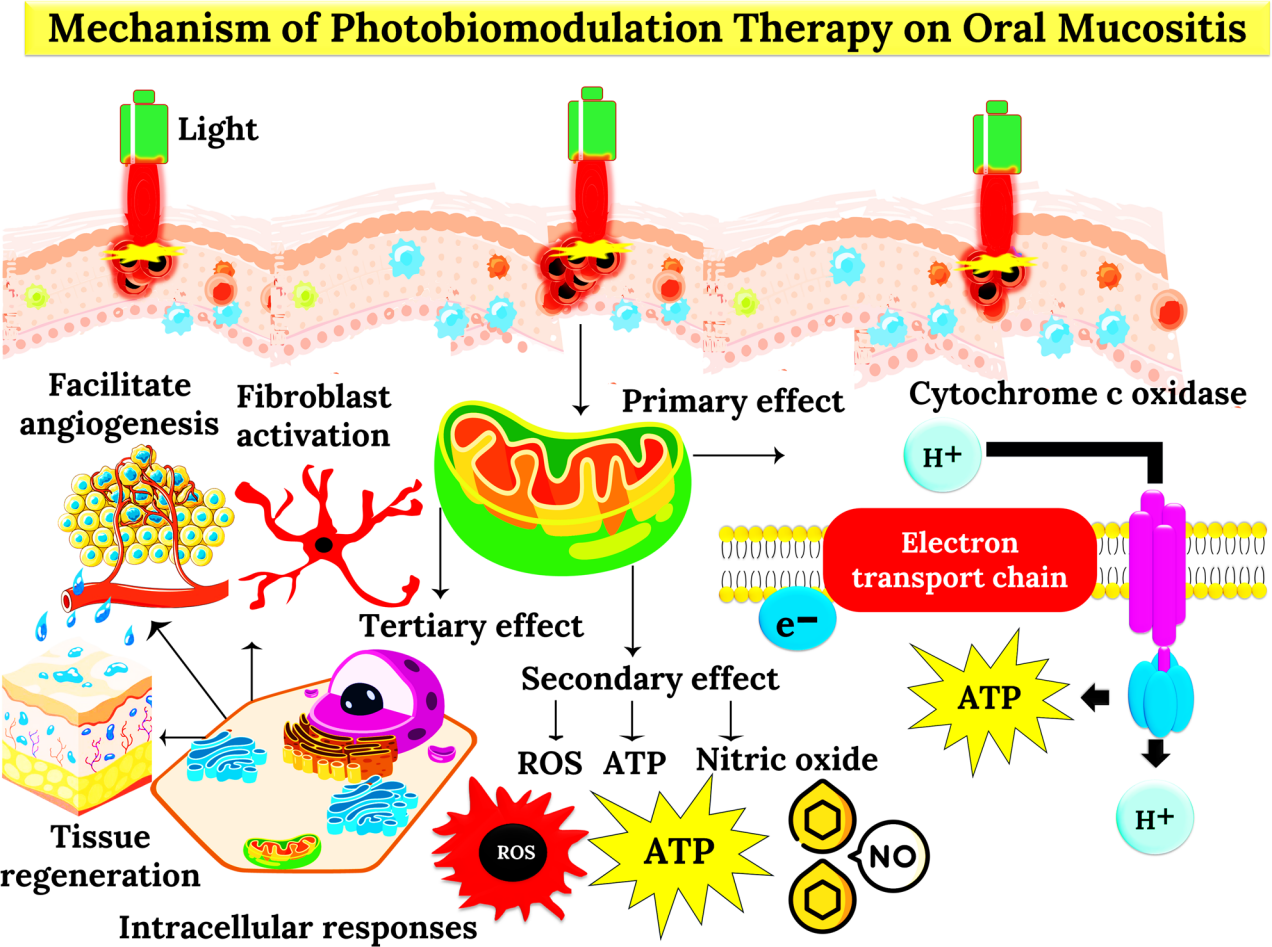
Mechanism of photobiomodulation therapy in oral mucositis. Low-level light penetrates the oral epithelium, targeting mitochondria and stimulating the electron transport chain. This increases proton (H⁺) gradients, enhances ATP synthesis, and modulates reactive oxygen species (ROS) and nitric oxide (NO) levels. The resulting bioenergetic and signaling changes promote cellular repair, reduce inflammation, support tissue regeneration, and accelerate the healing of mucosal lesions

Chemotherapy and radiotherapy, while central to cancer management, frequently induce severe mucosal and neural toxicities such as oral mucositis, dysgeusia, and peripheral neuropathy, all of which significantly compromise treatment adherence and quality of life. Conventional preventive strategies remain largely ineffective, prompting interest in non-invasive modalities that can promote tissue repair and mitigate inflammation without pharmacological burden. Photobiomodulation (PBM), involving the controlled application of low-intensity red or near-infrared light, has emerged as a promising supportive therapy owing to its ability to modulate mitochondrial activity, enhance cellular metabolism, and reduce oxidative stress. Recent randomized clinical trials across diverse oncologic populations have systematically examined the therapeutic potential of PBM in mitigating treatment-related complications. The following section synthesizes key studies that provide clinical and mechanistic insights into PBM’s efficacy, irradiation parameters, and evidence quality in the management of chemotherapy-and radiotherapy-associated adverse effects. The mechanism of PBM in oral mucositis is graphically represented in Fig. 5.

Reyad and colleagues conducted a randomized controlled clinical trial at Alexandria University Children’s Hospital, Egypt, to evaluate the efficacy of PBM in treating chemotherapy-induced oral mucositis (OM) among 44 children with acute lymphoblastic leukemia (ALL). The participants were randomly assigned to receive either conventional symptomatic care or PBM in addition to standard treatment. PBM was administered using a handheld diode laser (Dr. Smile Simpler) at 980 nm, 1.5 W, for 30 s per lesion, delivering an energy density of 4.5 J/cm² in continuous non-contact mode, across four consecutive daily sessions. The treatment targeted visible mucosal lesions on the basis of the WHO mucositis scale. The PBM group showed significantly reduced pain by day 10 and decreased mucositis severity by day 14 (*p* < 0.001 and *p* = 0.003, respectively), with no adverse events. This study demonstrated that PBM offers rapid symptomatic and morphological recovery in pediatric leukemic patients. While it was a single-center, open-label design without a sham control, adherence to CONSORT guidelines and use of validated pain and mucositis scales lend moderate-to-high evidence quality, confirming PBM as a safe, clinically effective adjunctive therapy for pediatric OM [87].

In line with the above findings, Khalil et al. investigated PBM as a preconditioning prophylaxis against chemotherapy-induced oral mucositis (OM) and xerostomia in a prospective, randomized, double-blind clinical trial involving 45 patients undergoing their first round of chemotherapy. The participants were divided into three groups: standard oral care, intraoral PBM, and combined intraoral + extraoral PBM. The intraoral arm received PBM using a 635 nm diode, 200 mW/cm², 20 s per point, 4 J/cm², applied to 24 intraoral sites (buccal, labial, tongue, floor, soft palate) in a single pre-chemotherapy session. The combined arm used the same intraoral protocol plus an extraoral 980 nm diode (200 mW/cm², 20 s per point, 4 J/cm²) at six facial landmarks. After one and two weeks, 73–86% of PBM patients remained OM-free, compared with universal OM occurrence in the control group (*p* < 0.001). The quality-of-life indices (OHIP-14) significantly improved (*p* < 0.05). The double-blind randomized design, adherence to ISRCTN registration (ISRCTN70634383), and precise irradiation parameters indicate high evidence quality, although the limited sample size and short follow-up reduce generalizability. Preconditioning with PBM thus appears to be a potent preventive approach for mucosal toxicity during chemotherapy [88].

In parallel, Ferreira et al. performed a randomized, placebo-controlled clinical trial to assess the role of PBM in mitigating gustatory dysfunction during hematopoietic cell transplantation (HCT). Eighty-five adult patients (42 PBM, 43 placebo) were randomized at the Hospital Israelita Albert Einstein, São Paulo. The PBM protocol used an InGaAlP diode laser (660 nm, 100 mW, 1.1 W/cm², 8 s/point), delivering 8.8 J/cm² at 12 points on the tongue dorsum, apex, circumvallate region, and lateral borders, administered daily from conditioning through neutrophil engraftment. A sham group underwent an identical procedure with the laser deactivated. Compared with placebo, PBM significantly reduced the incidences of hypogeusia, ageusia, and parageusia during neutropenic and engraftment periods, and prevented lingual papillae atrophy. The robust design, double-blind outcome assessment, daily intervention schedule, and clearly defined irradiation parameters confer high methodological quality. Minor limitations included the use of a single-site cohort and a lack of long-term follow-up. Overall, this trial provides strong evidence that PBM can preserve gustatory function and mucosal integrity during aggressive chemotherapeutic conditioning [89].

In a recent study, Elkady et al. performed a randomized, double-blind, sham-controlled clinical trial at Egypt’s Children’s Cancer Hospital 57,357, involving 42 children with acute myeloid leukemia (AML) receiving chemotherapy. The PBM arm received 660 nm diode irradiation (25 mW output, 0.25 J/point, 10 s per point) delivered once daily for five consecutive days (days 1–5 of chemotherapy) across 44 intraoral points, covering the buccal, labial, lingual, floor, and soft-palate sites, while controls received sham exposure. Clinical assessment using WHO and NCI CTCAE v4.0 scales revealed a significant reduction in OM incidence and severity (*p* < 0.001) across all follow-ups (days 5, 12, 19, and 30). Children in the laser group maintained healthy mucosa longer and experienced faster recovery when mild OM developed. The trial’s ethical rigor, pediatric focus, and double-blind design contribute to moderate-to-high evidence quality. Minor inconsistencies in reported power output (likely typographical) and limited follow-up slightly constrain reproducibility, but findings convincingly support the prophylactic use of PBM in pediatric AML chemotherapy [90].

Another study by Malta et al. conducted a phase II, randomized, triple-blind, placebo-controlled clinical trial with 112 breast cancer patients receiving adjuvant or neoadjuvant doxorubicin–cyclophosphamide (AC) chemotherapy. PBM was administered using a DMC Therapy EC laser at 660 ± 10 nm (red) and 808 ± 10 nm (infrared), each 100 mW, with a 0.098 cm² tip in contact mode. On each chemotherapy day (day 0) and day + 21 over four cycles, 2 J red light (20.32 J/cm²) for tissue regeneration and 3 J infrared light (30.48 J/cm²) for neuroprotection were applied to 14 symmetric points across the tongue dorsum. PBM significantly preserved objective and subjective taste perception (*p* < 0.05), maintained body weight, improved ECOG performance, reduced cachexia and gastrointestinal toxicity, and enhanced quality of life relative to placebo. The study’s meticulous laser dosing, triple blinding, and validated outcome metrics provide high-quality evidence. This technique establishes dual-wavelength PBM as a reliable, non-invasive intervention to prevent chemotherapy-induced dysgeusia and improve systemic well-being during AC therapy [91].

Pereira et al. compared PBM alone versus PBM + FITOPROT (a mucoadhesive mouthwash containing Curcuma longa and Bidens pilosa extracts) in 52 head and neck cancer patients undergoing radiotherapy. Both groups received PBM via a 660 nm InGaAlP diode, 25 mW, continuous contact mode, 10 s/point, 0.25 J per point (6.2 J/cm²), five days per week throughout the radiotherapy course. Each session included 40–50 intraoral sites, covering the buccal, labial, lingual, palatal, and sublingual mucosa, spaced ≥ 1 cm from the tumor site to ensure oncologic safety. The combination group gargled FITOPROT twice daily in addition to PBM. Although OM severity did not differ statistically significant between groups, FITOPROT + PBM modulated salivary nitrite and cytokine profiles (reducing TNF-α and IL-1β, increasing IL-10), suggesting an enhanced anti-inflammatory balance. This randomized trial, registered with ReBEC (RBR-9vddmr) and following CONSORT standards, demonstrates moderate-to-high evidence quality. This finding reinforces PBM as a standard preventive strategy for radiotherapy-induced mucositis and highlights the potential synergistic role of phytomedicines in cytokine regulation [92].

Teng et al. conducted a randomized, single-blind, sham-controlled phase II trial to evaluate PBM’s efficacy in managing established chemotherapy-induced peripheral neuropathy (CIPN) among 44 cancer survivors who had completed neurotoxic chemotherapy ≥ 3 months prior. The participants received either active PBM or sham treatment twice weekly for 6 weeks (12 sessions) using an Acupak CL Mini diode laser (658 nm, 8 mW, continuous). Irradiation targeted 16 points on hands and feet (interdigital spaces) plus 10 dermatomal sites (C6–T1 and L5–S1 bilaterally). The dose began at 1 J/point and was escalated to 2 J/point as tolerated. Compared with those at baseline, patient-reported symptom scores (FACT/GOG-Ntx13, EORTC QLQ-CIPN20) improved in both arms, but the laser group maintained sustained benefit at 12 weeks, whereas the controls regressed toward baseline. Adverse events were minimal and self-limiting. The trial provides moderate evidence quality, limited mainly by its small sample size and single-blinding; however, it presents promising evidence that low-intensity PBM may induce neurosensory recovery through peripheral and mitochondrial modulation [93].

Across these studies, rigorously conducted trials, PBM has demonstrated consistent efficacy and safety in alleviating or preventing oral mucositis, dysgeusia, and peripheral neuropathy across cancer populations, from pediatric leukemia and AML to adult breast and head-neck cancers. The wavelengths ranged from 635 to 980 nm, with power outputs between 25 mW and 1.5 W, and energy densities ranging from 0.25 to 30 J/cm², depending on indication and anatomical target. Daily or session-based irradiation over several days or weeks significantly reduced inflammation, pain, and tissue injury, alongside improved functional and quality-of-life outcomes. Collectively, the evidence quality ranges from moderate to high, with the strongest support for OM management and emerging validation for sensory neuropathies. These trials collectively strengthen the translational foundation for PBM as a cost-effective, non-invasive supportive therapy in oncology.

Collectively, the reviewed clinical trials demonstrate that PBM, applied within the red-to-near-infrared spectrum (635–980 nm) and at low power densities, can significantly reduce the incidence and severity of chemotherapy-induced oral mucositis, dysgeusia, and peripheral neuropathy, while improving patient comfort and functional outcomes. Its favorable safety profile, ease of administration, and reproducible biostimulatory effects make it an attractive adjunct to conventional oncology care. Future research should prioritize large, multicenter phase III trials with harmonized dosimetric parameters, standardized reporting frameworks, and long-term follow-up to validate the durability of response and cost-effectiveness. The integration of PBM with biomarker-guided or phytochemical approaches, as evidenced by recent combination protocols, may further optimize its clinical utility. Overall, PBM represents a biologically sound and clinically viable strategy poised to transform supportive care paradigms in oncology.

## Mechanistic insights into the immunoregulatory actions of photobiomodulation

**Fig. 6:**
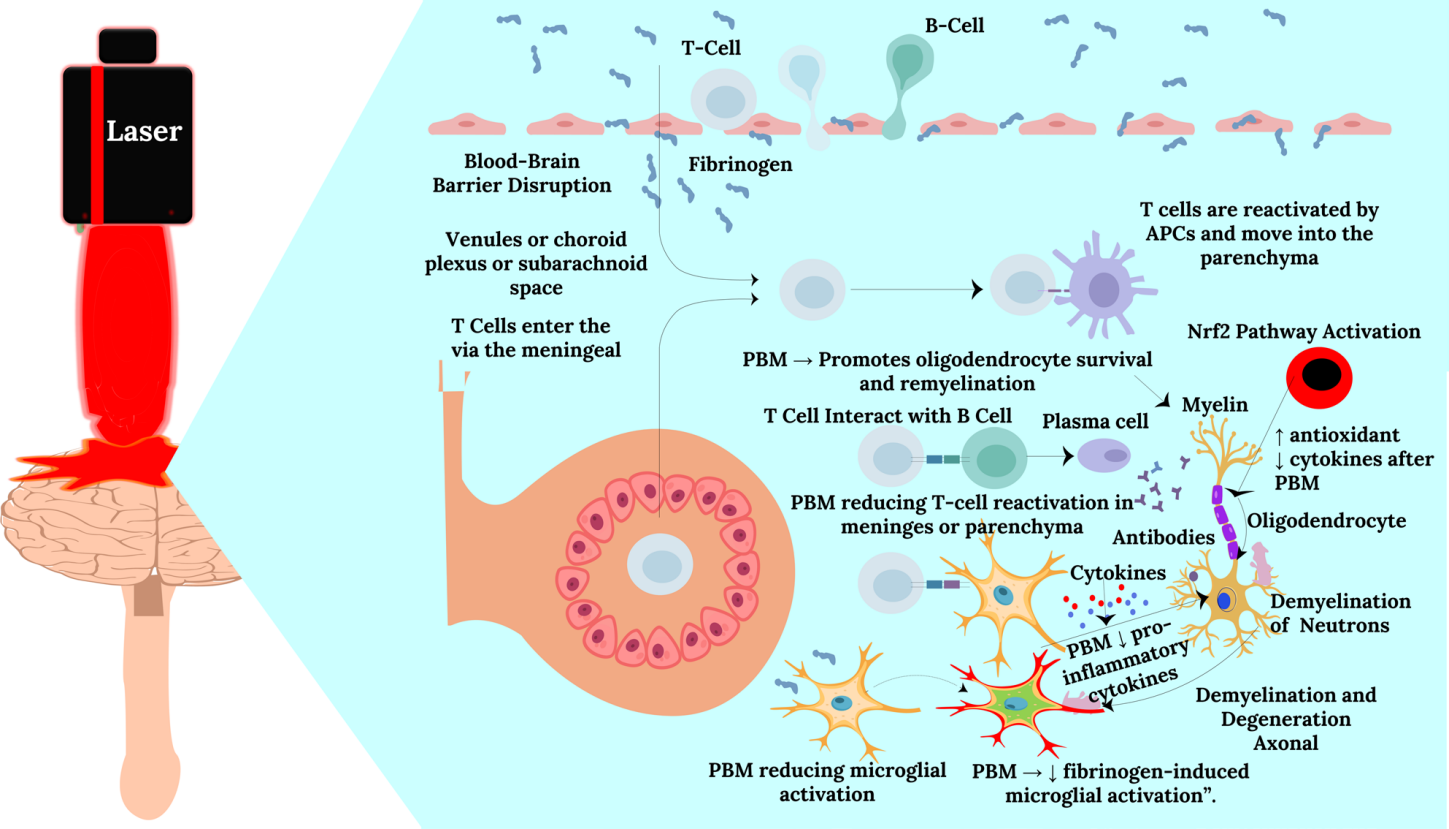
Immunomodulatory effects of photobiomodulation (PBM) in neuroinflammation. Schematic showing how PBM modulates immune responses within the central nervous system. PBM activates the Nrf2 pathway, reducing oxidative stress and pro-inflammatory cytokine release, suppressing T-cell reactivation, and attenuating fibrinogen-induced microglial activation. These effects collectively promote oligodendrocyte survival and protect against demyelination and axonal degeneration

Photobiomodulation therapy (PBMT), which employs low-power red to near-infrared (NIR) light, has emerged as a versatile biophysical approach that modulates cellular metabolism, redox homeostasis, and immune signaling through non-thermal mechanisms. By targeting chromophores such as cytochrome c oxidase within the mitochondrial respiratory chain, PBMT enhances ATP synthesis and initiates a cascade of downstream events involving the modulation of transcription factors, cytokine release, and oxidative stress responses. The unique advantage of this therapy lies in its ability to achieve systemic immunoregulatory and reparative effects without pharmacological toxicity, making it a promising adjunct in the management of autoimmune, neurodegenerative, and chronic inflammatory conditions [2]. Figure 6 schematically illustrates the capacity of PBMT to downregulate the expression of pro-inflammatory mediators (e.g., TNF-α, IL-1β, IL-6) while enhancing the expression of anti-inflammatory cytokines (e.g., IL-10), thus promoting cellular resilience and tissue homeostasis.

Autoimmune disorders represent a primary domain in which the immunomodulatory potential of PBMT has been most rigorously explored. In multiple sclerosis (MS), a prototypical autoimmune demyelinating disorder mediated by CD4⁺ Th1/Th17 responses, oxidative stress, and mitochondrial dysfunction exacerbates chronic neurodegeneration. Tolentino et al. conducted both in vitro and ex vivo analyses using peripheral blood mononuclear cells (PBMCs) and CD4⁺ T cells from MS patients and healthy donors. Their controlled design employed 670 nm, 735 nm, and 830 nm PBMT exposures under standardized conditions. The study reported wavelength-specific effects; 670 and 830 nm notably suppressed IFN-γ and elevated IL-10 levels, confirming a shift toward an anti-inflammatory phenotype. Importantly, these cytokine shifts were more pronounced in patient-derived cells, suggesting disease-specific immunosensitivity to photobiomodulatory cues. The methodological strength of this study lies in its dual-cell model validation (PBMCs and purified CD4⁺ subsets), supporting the reproducibility and translational potential of the observed cytokine modulation [94].

Complementing these human cell-based findings, Escarrat et al. performed a well-controlled preclinical investigation in an experimental autoimmune encephalomyelitis (EAE) model. Daily dorsoventral PBMT significantly improved motor and sensory functions. Using advanced two-photon longitudinal imaging in triple-fluorescent reporter mice, the authors demonstrated reduced glial activation, decreased leukocyte infiltration, and normalization of neuronal excitability in both the dorsal and ventral spinal regions. Electrophysiological and histopathological correlations confirmed that PBMT restored excitability homeostasis by attenuating neuroinflammatory hyperactivity. The study’s robust experimental design, including behavioral, cellular, and electrophysiological endpoints, provides high mechanistic fidelity, establishing PBMT as both an anti-inflammatory and neuroprotective agent in autoimmune demyelination [95]. Together [94] and [95] underscore a unified mechanism: PBMT ameliorates immune dysregulation by suppressing NF-κB–mediated cytokine expression and restoring redox balance, culminating in neurofunctional recovery.

In rheumatoid arthritis (RA), another systemic autoimmune disease characterized by chronic synovial inflammation and joint degradation, PBMT also has profound anti-inflammatory efficacy. Ryu et al. conducted a combined in vitro and in vivo study using fibroblast-like synoviocytes (FLSs) from RA patients and a collagen-induced arthritis (CIA) mouse model. RA-FLSs irradiated with 610 nm LED light (5–10 mW/cm²) exhibited reduced proliferation, migration, and invasion through suppression of NF-κB and NLRP3 inflammasome signaling pathways. In vivo PBMT (10 mW/cm², 40 min/day for two weeks) significantly decreased disease severity and systemic pro-inflammatory cytokine levels. The study included appropriate controls (non-irradiated and methotrexate-treated groups) and revealed synergism between PBMT and MTX, supporting its adjunctive therapeutic value. Mechanistically, PBMT reduces pannus formation and osteoclast activation by disrupting NF-κB–driven cytokine amplification, which translates directly to improved joint integrity and pain reduction. This evidence exemplifies how the molecular suppression of NF-κB–NLRP3 signaling aligns with clinically meaningful outcomes in autoimmune pathology [96].

Neurodegenerative diseases, particularly AD, are characterized by chronic neuroinflammation, mitochondrial dysfunction, and blood–brain barrier (BBB) breakdown. The photobiological effects of PBMT on mitochondrial and endothelial integrity make it a compelling candidate for neurovascular repair. In a meticulously designed preclinical study, C. Ma et al. treated APP/PS1 transgenic mice with 808 nm PBMT (20 mW/cm²) for six weeks. Behavioral assays confirmed cognitive improvement and reduced anxiety-like behaviors. Mechanistically, PBMT enhanced tight-junction protein expression (Occludin, Claudin-5, ZO-1) and promoted Aβ clearance via LRP1-mediated transport and microglial phagocytosis. Mitochondrial assays showed reduced oxidative stress and apoptosis, while activation of the AMPK pathway emerged as the central mediator of these effects. The use of AMPK inhibitors abrogated PBMT’s benefits, directly linking energy metabolism regulation to BBB protection. The strength of this study lies in its multimodal design and behavioral, biochemical, and mechanistic analyses, which provide high translational validity [97].

Building upon this neuroimmunological framework, Wu et al. demonstrated that PBMT enhances hippocampal neurogenesis and cognitive recovery in APP/PS1 and triple-transgenic (3xTg) AD mouse models by modulating T-cell–derived neurotrophic signaling. PBMT (10 J/cm² daily) activated the JAK2/STAT4/STAT5 cascade in CD4⁺ T lymphocytes, enhancing IL-10, IFN-γ, and TGF-β1 expression while upregulating IGF-1 and BDNF within the hippocampal microenvironment. Importantly, conditioned media from PBMT-treated T cells promoted neural stem cell differentiation in vitro, confirming an immune–neural interaction mechanism. The study combined behavioral, immunological, and molecular endpoints, indicating strong experimental reproducibility and relevance to human disease mechanisms [98]. These findings, together with [97], reveal a broader theme: PBMT counteracts ageing-associated neurodegeneration through coordinated regulation of mitochondrial metabolism, cytokine balance, and neurotrophic signaling, reinforcing its role in age-related disease mitigation.

Inflammatory macrophage activation constitutes a central barrier to tissue repair in spinal cord injury (SCI) and chronic inflammatory disorders. Y. Ma et al. employed an implantable biofiber-optic PBMT system (continuous delivery for four weeks) in a murine SCI model, achieving precise irradiation and longitudinal assessment. PBMT selectively inhibited M1 macrophage polarization and associated cytokines (IL-1α, IL-6) while sparing M2 populations. Transcriptomic and proteomic analyses revealed suppression of Notch1–HIF-1α/NF-κB signaling, correlating with reduced neurotoxicity and improved motor recovery. The comprehensive design, including in vitro macrophage models and RNA sequencing validation, lends high confidence to the mechanistic conclusions [99].

Similarly, Zuo et al. investigated whether PBMT-induced autophagy mediates macrophage modulation post- spinal cord injury (SCI). Using a clamped SCI model, mice received PBMT for 28 days, resulting in substantial functional recovery. Western blot and immunofluorescence analyses showed that increased levels of autophagy markers (LC3, Beclin-1) were markedly downregulated and whereas inflammasome-related proteins (NLRP3, Caspase-1, and IL-1β) were significantly upregulated. Bioinformatic integration identified Toll-like receptor 2 (TLR2) as the central regulatory node in PBMT-mediated autophagy modulation. Pharmacological activation of TLR2 reversed the effects of PBMT, confirming its pathway specificity. The dual in vivo and in vitro validation and RNA-seq integration exemplify methodological rigor and mechanistic depth [100].

A complementary mechanistic layer was revealed by Tian et al., who investigated the effects of PBMT (980 nm, 0.5–10 J/cm²) on RAW264.7 macrophages in a controlled in vitro model of periodontitis-related inflammation. PBMT enhanced PI3K/AKT/mTOR signaling and induced M2 polarization, increasing IL-10 and Arg1 while suppressing TNF-α, IL-1β, and iNOS expression. Inhibition of PI3K by LY294002 abolished these effects, confirming causal linkage. These studies collectively define a multi-axis immunoregulatory mechanism whereby PBMT suppresses pro-inflammatory (NF-κB, TLR2, and Notch1–HIF-1α) signaling while enhancing metabolic repair pathways (PI3K/AKT/mTOR, autophagy), orchestrating macrophage reprogramming and tissue regeneration [101].

In localized autoimmune or chronic mucosal inflammatory diseases, PBMT has demonstrated clinical and molecular efficacy in promoting epithelial regeneration and symptom relief. Mutafchieva et al. conducted a prospective clinical trial involving 20 patients with oral lichen planus (OLP), using an 810 nm diode laser (0.5 W, 1.2 J/cm², 3× weekly for one month). Clinical outcomes revealed significant reductions in pain, Visual Analogue Scale (VAS), and lesion size, accompanied by histological evidence of increased bcl-2 and Ki-67 expression, indicating enhanced anti-apoptotic and proliferative activity. Erosive forms of OLP responded more favorably, suggesting that PBMT is particularly beneficial for high-inflammatory subtypes. The strength of this study lies in its combined clinical–molecular evaluation with matched controls, providing a clear mechanistic link between oxidative stress modulation and epithelial repair [102].

In a mechanistic complement, Pasternak-Mnich et al. explored dual-wavelength PBMT (808 + 905 nm) effects on human PBMCs, comparing cumulative and fractionated regimens across multiple frequencies. The controlled in vitro design demonstrated the selective induction of IL-1β, CCL2, and CCL3 transcripts under defined irradiation parameters, with transient increases in protein levels. While initially pro-inflammatory, these effects are hypothesized to represent a preconditioning phase that primes immune cells toward subsequent anti-inflammatory resolution. Despite being limited to cell-based assays, this study underscores the dose- and frequency-dependence of PBMT responses, supporting the need for standardized irradiation protocols in clinical translation [103].

Beyond localized lesions, PBMT exerts systemic immunoregulatory effects relevant to neuroinflammation. Shamloo et al. used a robust in vivo mouse model to assess the ability of PBMT to counteract lipopolysaccharide (LPS)-induced systemic and central inflammation. The mice received daily red/NIR light exposure for ten days, with or without 40 Hz gamma-frequency flicker. Both modalities suppressed the inflammasome-associated cytokines IL-1β and IL-18 while increasing the level of IL-10. The gamma-PBMT combination showed synergistic enhancement, highlighting potential coupling between photonic and neural oscillatory modulation. The use of systemic inflammatory models and the quantification of both peripheral and central cytokine responses strengthen the translational relevance of these findings, suggesting therapeutic applicability in sepsis-associated or neuroinflammatory conditions [104].

Collectively, evidence from these studies delineates PBMT as a multi-target, systems-level modulator of immune and repair pathways. Across autoimmune (MS, RA), neurodegenerative (AD), and inflammatory (SCI, OLP, periodontitis) models, PBMT consistently converges on redox-sensitive signaling hubs such as NF-κB, MAPK, and HIF-1α. These upstream regulators orchestrate downstream modulation of cytokine networks, suppress IL-1β, IL-6, and TNF-α, and enhance IL-10 and TGF-β1, thereby rebalancing pro- and anti-inflammatory states. PBMT therapy also enhances energy metabolism through AMPK and PI3K/AKT/mTOR activation, augments autophagic clearance, and promotes neurotrophic signaling (BDNF, IGF-1), collectively translating into reduced inflammation, restored tissue integrity, and improved function.

In terms of evidence quality, the reviewed studies encompass a balanced spectrum of in vitro mechanistic analyses, preclinical animal models, and early-stage clinical investigations. Studies such as [95]; [96]; [97]; and [99] demonstrate high experimental rigor, employing appropriate controls, blinding, and multimodal analyses, thereby offering strong preclinical evidence (Level II). Clinical studies like [102], although limited in cohort size, provide Level III human evidence with objective clinical endpoints and molecular correlates. Conversely, studies like [103], while insightful, are limited by their in vitro scope and warrant replication under physiologically relevant conditions. Collectively, these findings present a consistent mechanistic consensus with moderate-to-high translational validity, although standardized PBMT protocols are needed for interstudy comparability.

From an integrative standpoint, PBMT’s actions can be conceptualized as a unifying photoimmunometabolic modulation process, wherein mitochondrial activation initiates downstream control over redox signaling, cytokine balance, and cellular differentiation. This convergence explains the broad-spectrum efficacy of PBMT across autoimmune, age-related, and inflammatory diseases. In autoimmune models (MS, RA), PBMT primarily acts through suppression of NF-κB and NLRP3; in neurodegeneration, through AMPK and JAK/STAT activation; and in injury models, through Notch1–HIF-1α, PI3K/AKT/mTOR, and autophagy regulation. Linking these mechanisms to clinical phenomena, such as pain reduction, neuronal recovery, and epithelial regeneration, reinforces PBMT’s translational promise. However, challenges remain. Evidence heterogeneity persists due to inconsistent reporting of irradiation parameters (wavelength, fluence, duty cycle), small human cohorts, and limited long-term follow-up. Future research must emphasize randomized controlled clinical trials, multi-omics mechanistic analyses, and standardized dosimetry to strengthen causal inference. Furthermore, exploration of combinatorial PBMT with pharmacologic or rehabilitative interventions (e.g., stem cell therapy or neuromodulation) could amplify therapeutic outcomes.

Synthesizing the collective evidence from [94] through [100], PBMT has emerged as a mechanistically coherent, multi-level therapeutic approach capable of simultaneously regulating immune, oxidative, and regenerative processes. Its ability to modulate the NF-κB, MAPK, AMPK, and JAK/STAT signaling pathways bridges molecular and clinical outcomes across autoimmune, neurodegenerative, and inflammatory conditions. The convergence of mitochondrial bioenergetics enhancement, cytokine rebalancing, and immune cell reprogramming defines PBMT as a truly integrative, non-pharmacological therapeutic paradigm for age-related and autoimmune pathologies. With robust mechanistic underpinnings and growing translational validation, PBMT stands poised to transition from adjunctive therapy to a mainstream modality in precision photomedicine.

## Treatment parameters and optimization in photobiomodulation

Photobiomodulation (PBM) has emerged as a powerful non-invasive therapeutic modality capable of modulating cellular metabolism, stimulating collagen synthesis, and promoting wound healing through light-induced biochemical reactions. Despite its promising outcomes across dermatological, dental, neurological, and rehabilitative applications, a major limitation hindering its clinical translation is the lack of standardized treatment protocols. Considerable variability exists in wavelengths, power densities, fluences, irradiation durations, and session frequencies across studies. This heterogeneity not only complicates inter-study comparison but also contributes to inconsistent clinical outcomes. An analytical review of recent randomized controlled trials highlights both the therapeutic versatility of PBM and the urgent need to establish optimized, reproducible, and evidence-based clinical parameters.

In aesthetic dermatology, Bragato et al. conducted a carefully standardized investigation of red LED PBM (660 nm, 6.4 mW/cm², 8.02 J/cm², 5.02 mW, 21 min per session) aimed at promoting facial rejuvenation in women aged 45–60 years. By comparing treatment frequencies, twice versus three times weekly over four weeks, against a simulated control, this study directly addressed how exposure frequency modulates efficacy, a variable often overlooked in earlier PBM studies. Objective wrinkle assessments using ImageJ-based analysis and optical coherence tomography, alongside subjective satisfaction scales, yielded quantitative and perceptual measures of skin improvement. Importantly, this work exemplifies how methodological precision, fixed wavelength, controlled fluence, consistent exposure time, and sham controls can minimize the variability that has historically undermined PBM reproducibility. However, the broader literature remains inconsistent, with studies using red, near-infrared, and even multiwavelength LED combinations without a standardized rationale, highlighting the need for protocol harmonization [105].

In dental applications, the analgesic potential of PBM has been widely explored, yet parameter diversity remains a major issue. Tolentino et al. carried out a randomized, double-blind, controlled trial comparing three protocols: 3% potassium nitrate gel, PBM at 808 nm (1 J per point, 0.028 cm² spot), and a combination of both, in treating cervical dentin hypersensitivity (CDH). Although all interventions achieved statistically significant and long-lasting pain reduction for up to three months, the absence of differences between groups suggests that the effect of PBM may plateau beyond a threshold fluence or dosage. This insight underscores the necessity of delineating dose–response relationships, a gap that persists across much of the PBM literature. Despite the rigorous study design, CONSORT adherence, sham simulations, and randomized allocation, the limited discussion of irradiance variability or tissue optical absorption coefficients reveals how incomplete reporting of dosimetric details hampers comparability and reproducibility in clinical PBM research [106].

Similarly, Miranda et al. applied a triple-blind, placebo-controlled design to examine the efficacy of LED PBM (940 nm, 4 J/cm², 160 mW, 10 min) in reducing bleaching-induced dental sensitivity. The study demonstrated that pre-treatment with PBM significantly decreased both the frequency and intensity of sensitivity without compromising whitening results. While methodologically robust, employing both VITA shade guides and spectrophotometric measurements, the study’s relevance to clinical optimization lies in its demonstration that the timing of PBM application, in this case, preconditioning, plays a crucial role in patient comfort. However, variability persists across similar studies, where wavelength choices range from 810 nm to 970 nm and fluences from 1 to 10 J/cm², often without standardized justification. This inconsistency in clinical parameters contributes to a fragmented evidence base and limits the formulation of universal PBM treatment protocols [107].

The influence of application duration as a dose-modulating factor was explored by Elbay et al. in a prospective, randomized, triple-blind trial evaluating injection pain during dental anesthesia in children aged 6–12 years. Using a 940 nm diode laser at 0.3 W for 20, 30, or 40 s (energy densities of 69, 103, and 138 J/cm²), the authors reported a nonsignificant trend toward reduced pain with longer exposure times. While the statistical insignificance might suggest limited effect, this study contributes important insight into parameter sensitivity, the idea that PBM outcomes depend heavily on the interplay of energy density, exposure time, and target tissue characteristics. Moreover, it exemplifies exemplary evidence quality through its triple blinding, placebo simulation, and dual pain assessment scales (FLACC and PRS). However, as with many pediatric PBM trials, the lack of long-term follow-up and incomplete reporting of tissue absorption or optical depth data remain limitations that impede reproducibility [108].

For systemic applications, Chang et al. conducted a randomized, single-blind, placebo-controlled trial to test whether 830 nm PBM could improve sleep quality and quality of life (QoL) in end-stage renal disease patients undergoing hemodialysis. By targeting both the palm and acupuncture points (KI1 and ST36) with defined energy densities (256.10 and 109.76 J/cm²), the study demonstrated significant improvements in the Pittsburgh Sleep Quality Index (PSQI), Athens Insomnia Scale (AIS), and QoL metrics. The methodological rigor, precisely defined dosimetry, validated questionnaires, and controlled exposure sites provide a model of reproducibility. However, the brief intervention duration (one week) highlights a broader issue of protocol variability in treatment frequency and follow-up periods across PBM research. Some studies administer daily sessions, while others rely on weekly exposures, complicating meta-analytic synthesis and clinical guideline formulation. This trial’s robust design strengthens the evidence base supporting PBM’s neuromodulatory potential but also underscores the urgent need for standardized reporting of exposure frequency, dose per session, and cumulative energy, which are critical determinants of long-term therapeutic effects [109].

Evidence of PBM’s systemic and rehabilitative benefits was further reinforced by Neto et al., who evaluated low-intensity red (635 ± 10 nm) and near-infrared (880 ± 20 nm) LED therapy in critically ill ICU patients. The randomized, triple-blind design and the use of a neoprene “LED blanket” delivering 2,073.6 J per session represent methodological strengths rarely matched in clinical PBM studies. The intervention resulted in an ICU stay of approximately 30% (*p* = 0.028), improved mobility by 255% vs. 110% in controls (*p* = 0.007), enhanced MRC muscle strength by 12% vs. a 9% decline in the sham group (*p* = 0.001), and increased handgrip strength by 34% vs. a 13% decrease (*p* < 0.001), without altering SAPS 3 scores between groups. Importantly, this trial highlights the effects of energy distribution and total dose delivery across multiple anatomical sites, demonstrating that spatial coverage and total irradiance are as important as wavelength in determining clinical efficacy. The rigor of blinding and inclusion of objective performance outcomes (MRC score, IMS, handgrip) elevate this trial’s evidence grade. Nevertheless, inconsistencies in total energy reporting among similar ICU trials point to a broader methodological gap: a lack of uniform energy normalization metrics across different body regions and device configurations [110].

The economic and translational implications of PBM were rigorously explored by Jana Neto et al. in a randomized, double-blind trial examining the cost-effectiveness of LED PBM for soft-tissue healing in tibial fractures. Using a tri-wavelength approach (420, 660, 850 nm; 3 J per point; 10 min sessions), the study reported accelerated wound resolution (13.1 vs. 23.1 days) and substantial cost savings (R$7,527 per patient). In addition to demonstrating clinical efficacy, this study was exceptional in integrating economic outcome modeling, a dimension often neglected in PBM literature. The meticulous inclusion of direct and indirect costs (hospitalization, materials, labor, productivity) provided a comprehensive understanding of the translational value of PBM. However, it also revealed inconsistencies in the reporting of fluence per wavelength and energy distribution uniformity, echoing the broader problem of insufficient protocol transparency [111].

Similarly, Jana Neto et al. conducted another double-blind, placebo-controlled trial in which a multiwavelength LED PBM (420, 660, 850 nm; 432 J total per session) was applied to manage soft-tissue injuries in tibial and ankle fractures. The results confirmed significant acceleration of wound healing, reduced pain, and a fourfold decrease in infection rates compared with those in the sham treatment group. The daily application protocol and stratification by wound size ensured methodological precision and clinical relevance. Importantly, the absence of adverse reactions and the use of the validated Bates–Jensen Wound Assessment Tool add strength to evidence quality. However, when contextualized alongside similar studies, marked differences in energy per diode, treatment duration, and number of sessions again illustrate the lack of a universal dosing standard, a key contributor to inter-study variability and inconsistent reproducibility [112].

Taken together, these studies collectively underscore both the promise and the challenges of PBM optimization. Across diverse clinical contexts, from facial rejuvenation to ICU rehabilitation, the therapeutic response consistently depends on precisely tuned photonic parameters. Variability in wavelength (420–940 nm), fluence (3–256 J/cm²), power density (5–160 mW/cm²), and treatment frequency (daily to weekly) remains the major determinant of inconsistent outcomes. From an evidence-quality standpoint, most included trials demonstrate high methodological rigor, randomized, double- or triple-blind designs, validated measurement tools, and ethical registration, yet gaps persist in uniform reporting of dosimetric and procedural details. These omissions limit the aggregation of high-level evidence and preclude the formulation of consensus guidelines. Furthermore, protocol variability, spanning session duration, total energy, application site, and follow-up interval, remains inadequately discussed across studies, despite being key factors influencing PBM efficacy. Future research should prioritize protocol standardization frameworks, dose–response calibration studies, and the inclusion of biophysical biomarkers (such as mitochondrial enzyme activation or cytokine modulation) to bridge mechanistic and clinical evidence.

## Methodological and biological determinants of null outcomes in photobiomodulation therapy

Despite accumulating evidence that photobiomodulation therapy (PBMT) can enhance mitochondrial function, modulate inflammation, and promote neuroprotection, several rigorously controlled studies have reported null or inconclusive outcomes. These findings underscore that PBMT efficacy is highly dependent on a complex interplay of biophysical, physiological, and experimental factors. Parameters such as wavelength, irradiance, fluence, energy distribution, and timing of application critically determine whether the delivered dose falls within the therapeutic window necessary to elicit biological effects. Furthermore, participant or model characteristics, including training status, developmental stage, and disease severity, can introduce ceiling effects that mask subtle photobiological responses. This section synthesizes recent controlled human and preclinical studies reporting non-significant PBMT outcomes, emphasizing how variations in dosimetry, tissue penetration, and biological context shape experimental results and interpretability.

Azuma and colleagues conducted a double-blind, randomized, crossover trial to evaluate whether pre-exercise PBMT could enhance the muscular performance of the elbow flexors in untrained women. Thirteen participants received either active PBMT (808 nm AsGaAl laser) or a placebo 20 min before six sets of concentric elbow flexion repetitions were performed until failure at 60% of the one-repetition maximum. PBMT was applied to four stationary points over the biceps brachii at 100 mW output, delivering 7 J per point (total 28 J per arm; fluence ≈ 250 J/cm²). The study reported no differences between active and placebo treatments in the number of repetitions, perceived exertion, or delayed-onset muscle soreness (DOMS) up to 72 h post-exercise [113].

The lack of improvement can be attributed to several methodological and physiological factors. The energy density was likely excessive for the small muscle volume, potentially surpassing the optimal biphasic dose window where stimulatory effects plateau or reverse. Additionally, the use of four small stationary beams produced uneven energy distribution across the muscle, leaving parts under- or over-exposed. Biological ceiling effects may also have played a role; untrained female participants often demonstrate higher fatigue resistance and lower muscle damage in isolated arm exercises, limiting the detectability of PBMT-induced protection. Ovea, this study underscores the importance of dose optimization and beam geometry in muscle-level PBMT applications [113].

A randomized, placebo-controlled trial investigated the effects of PBM applied during a six-week (12-session) combined training program involving sprints and explosive squats on clinical, functional, psychological, and systemic outcomes in trained healthy males compared to placebo and passive recovery controls. Thirty-nine participants (aged 18–30 years; BMI 23.9 ± 3 kg/m²) were randomized into active PBM (30 J per site), placebo, or control groups. To minimize the confounding influence of initial neuromuscular adaptation, all participants completed a six-week pre-training phase before intervention. The PBMT device combined infrared light (875 nm LEDs, four emitters) with a 35 mT magnetic field, delivering approximately 3.99 J per site over a 20 cm² aperture (total energy ≈ 180 J; power density 19.44 mW/cm²). Outcome measures, including maximal voluntary isometric contraction (MVIC), squat jump performance, and vascular endothelial growth factor (VEGF) levels, were assessed at baseline and after six weeks. Analysis revealed no significant time-by-group interaction or group effect; however, a significant main effect of time was observed for MVIC (mean difference = 22 Nm/kg; 95% CI: 3.9–40) and squat jump height (mean difference = 1.6 cm; 95% CI: 0.7–2.5). No other outcomes demonstrated significant changes [114].

Several protocol limitations can explain the null result. The per-site energy was very low relative to muscle volume, and the diffuse, non-collimated LED output likely restricted effective penetration to superficial layers. The irradiance (~ 19.44 mW/cm²) was also below the empirically defined therapeutic window for deep muscle stimulation. Furthermore, the use of trained individuals may introduce ceiling effects wherein trained participants, already exhibiting maximal mitochondrial function, as well-conditioned muscles already possess high oxidative capacity and rapid recovery kinetics. Finally, PBMT was delivered post-exercise or during recovery periods; if the ergogenic mechanism relies on pre-exercise mitochondrial priming, the chosen timing could have missed the optimal bioenergetic window. These aspects collectively illustrate the interplay between irradiance, geometry, and participant characteristics in determining PBMT efficacy [114].

Flores et al. evaluated whether photobiomodulation has ergogenic effects on anaerobic performance in well-trained cyclists. Fifteen healthy male road and mountain bike cyclists participated in a randomized, double-blind, placebo-controlled crossover trial. Each athlete received either PBM (630 nm, 4.6 J/cm², 6 J per point, 16 points) or a placebo (PLA) treatment in the first session, followed by a 30-second Wingate test to assess the mean and peak power, relative power, mean and peak velocity, mean and peak RPM, fatigue index, total distance, time to peak power, explosive strength, and power drop. After a 48-hour washout period, the participants underwent the alternate intervention. The data were analyzed using repeated-measures ANOVA with Bonferroni post hoc or Friedman test with Dunn’s post hoc test (*p* < 0.05), and effect sizes were calculated using Cohen’s d. No significant differences (*p* >0.05) were observed between the PBM and PLA sessions for any performance variable. Only small effect sizes were found for time to peak power (-0.40; 1.11 to 0.31) and explosive strength (0.38; -0.34 to 1.09). These findings suggest that red light irradiation at low energy density does not produce ergogenic benefits for anaerobic cycling performance [115].

The absence of enhancement is consistent with two principal limitations. First, 630 nm red light results in limited tissue penetration and thus is unlikely to reach the deeper regions of the quadriceps and hamstrings engaged during high-intensity cycling. Near-infrared wavelengths (≈ 810 nm) penetrate more effectively and have shown greater bioenergetic activation and improved locomotor activity as demonstrated in other studies [116]. Second, the selected per-site fluence (4.6 J/cm²) and point-wise application pattern probably provided insufficient total energy to large muscle groups, producing heterogeneous irradiation. As the subjects were already highly trained, the physiological potential for further enhancement of mitochondrial or contractile performance was minimal [115]. Therefore, the null findings primarily reflect wavelength-specific limitations and ceiling effects, highlighting that red-light PBMT may be suboptimal for deep muscular applications in elite athletes.

Gutiérrez-Menéndez et al. investigated the effects of PBM on the prefrontal cortex and hippocampus of 23-day-old healthy male (*n* = 31) and female (*n* = 30) Wistar rats. Each sex was divided into three groups: a PBM-treated group (5 days of PBM), a device control group exposed to identical conditions without light, and a basal control group. The PBM groups received 36 cycles of PBM, with a total irradiation time of 24 min and an average fluence of 46.5 J/cm^2^ per day. Brain metabolic activity and immediate early gene activation were assessed using CCO histochemistry and cellular proto-oncogene Fos (c-Fos) immunostaining, respectively. Results revealed no significant metabolic or c-Fos expression differences among the groups in either sex, indicating that PBM had no measurable impact on the brains of young healthy rats under the tested conditions. Unlike findings in adult subjects, these results suggest that in developing brains, where mitochondrial function is already optimal, the effects of PBM on mitochondrial activity and neuronal activation may be undetectable through CCO and c-Fos analyses [117].

The absence of observable PBM effects in this study may be attributed to several methodological and biological factors. First, the use of healthy, developing rats likely limits the capacity to detect improvements, as their baseline mitochondrial and neuronal functions are already optimal, leaving minimal scope for enhancement. Additionally, the PBM parameters applied, such as irradiance, exposure duration, and beam geometry, may not have been optimal for targeting deep brain structures like the hippocampus and prefrontal cortex, resulting in insufficient light penetration or subtherapeutic dosing. The lack of effect could also reflect tissue-specific responsiveness, as developing neural tissue may differ from mature brain tissue in terms of light absorption and metabolic activation. Future studies should refine PBM protocols by systematically varying dosimetry and optical parameters to better delineate effective conditions for modulating brain function during early development [117].

Sipion et al. investigated the long-term effects of PBM on Alzheimer’s disease (AD) using behavioral and histological assessments in a well-characterized transgenic AD mouse model (5xFAD). The experiment was conducted as a randomized, fully blinded, and Good Laboratory Practice (GLP)-compliant in vivo study. Beginning at one month of age, the mice received cranial illumination with either no light (sham), low-power, or high-power 810 nm light three times per week for five months, low power PBM treatment (6 mW/cm^2^, *n* = 20) or high power PBM treatment (600 mW/cm^2^, *n* = 21), corresponding to the prodromal phase of AD pathology. Behavioral performance was evaluated using the Morris water maze, novel object recognition, and Y-maze tests, while histological analyses were used to assess amyloid burden, neuronal integrity, and microglial activation. Across all parameters, no significant differences were observed between the treatment groups. These findings indicate that, under the conditions tested, PBM did not exert measurable therapeutic benefits in this AD model [118].

The lack of observable therapeutic effects of PBMT in this study may be attributed to several methodological and biological factors that warrant consideration. One potential reason is the suboptimal dosing parameters, including irradiance and total energy delivered to the target tissue, which might have been insufficient to elicit a measurable neuromodulatory response. The beam geometry and light penetration depth at 810 nm could also have limited the effective delivery of photons to deeper brain regions implicated in Alzheimer’s pathology, such as the hippocampus and cortex. Furthermore, treatment during the prodromal stage of disease, when neuronal dysfunction may not yet be accompanied by extensive degeneration, could have reduced the likelihood of detecting significant improvements. The healthy baseline of the animals at treatment onset may therefore have masked subtle neuroprotective effects [118].

Collectively, the reviewed studies highlight that null PBMT findings are not indicative of inefficacy per se but rather of suboptimal parameterization relative to tissue type, physiological state, and disease context. Discrepancies in wavelength penetration (e.g., 630 nm vs. 810 nm), fluence distribution, and treatment timing underscore the need for standardized dosimetric frameworks and individualized dose calibration. Moreover, biological ceiling effects in healthy or highly trained subjects, and developmental or prodromal stages with limited dysfunction, can confound measurable outcomes. Future research should adopt precision photobiomodulation approaches that integrate computational light propagation models, quantitative spectroscopy-based dose monitoring, and adaptive trial designs to map the true therapeutic window across tissues and conditions. Establishing such mechanistic and methodological rigor will be essential for translating PBMT from experimental promise to reproducible clinical efficacy.

## Challenges and future research directions

Although photobiomodulation therapy (PBMT) has demonstrated efficacy across preclinical settings and targeted clinical contexts, reproducibility remains inconsistent due to methodological variability. Disparities in study design, including small cohort sizes, diverse disease models, and heterogeneity in baseline physiology, undermine cross-trial comparability [57]. For example, while PBMT enhanced post-surgical recovery in tendinopathies and musculoskeletal repair [56], other trials using similar wavelengths reported negligible benefits in trained individuals or healthy volunteers [114]. These inconsistencies frequently originate from differences in fluence (energy density), pulse frequency, and cumulative exposure, which critically influence mitochondrial excitation thresholds.

Furthermore, limited treatment blinding and insufficiently standardized sham controls compromise outcome reliability. The strong placebo component inherent in light-based interventions, suggested in randomized sham-controlled trials in patients with fibromyalgia and dermatitis [46], highlights the need for improved methodological rigor. The lack of uniform reporting standards for irradiation geometry, tissue depth penetration, and optical power calibration exacerbates inter-study discrepancies [4]. Comprehensive reporting templates, akin to CONSORT-PBM guidelines, should be mandated to ensure reproducibility across laboratories.

At the mechanistic level, PBMT is primarily understood to photostimulate cytochrome c oxidase within the mitochondrial respiratory chain, thereby enhancing ATP production and redox homeostasis [5]. However, beyond this canonical mechanism, evidence indicates a complex interplay of nitric oxide release, reactive oxygen signaling, and calcium-dependent transcriptional regulation that varies temporally and spatially across tissues [2, 6]. Neurodegenerative models show PBM-mediated improvements in mitochondrial gene expression and attenuation of synaptic degeneration [7, 8], yet analogous studies in inflammatory or vascular tissues reveal opposite dose-response relationships, suggesting that the biological context heavily dictates outcomes [104].

Recent findings indicate that the effects of PBMT depend on the baseline cellular metabolic status. Under oxidative, inflammatory, or hypoxic stress, cells exhibit heightened responsiveness [100], whereas normometabolic tissues may display negligible or biphasic responses, a hallmark of hormetic effects [117]. This dualism challenges the notion of universal therapeutic dosing and stresses the importance of adaptive, patient-specific dosing algorithms. Mechanistic biomarkers correlating energy absorption with mitochondrial, redox, and immune signaling remain underdeveloped, limiting translational predictability [103].

The formulation of universally valid PBMT protocols is hindered by a lack of standardized dosimetry. The published fluence ranges (0.1–60 J/cm²) vary greatly across tissues, with divergent power densities [11] and wavelengths spanning 630–1060 nm depending on the application site [17]. Some dermatologic and ophthalmologic trials demonstrate benefits at sub‑therapeutic fluences, whereas musculoskeletal applications demand deeper penetration via infrared wavelengths [11, 13]. This inconsistency complicates clinical adoption and suggests that tissue optical properties, chromophore distribution, and vascular perfusion dynamics must be integrated into dosimetric modeling.

Equally critical is the identification of biomarkers that reliably capture photobiological response. To date, PBMT studies have utilized disparate surrogate markers, mitochondrial membrane potential, serum cytokines, BDNF levels, or imaging-detected perfusion changes, with limited reproducibility [16]. The integration of metabolomic profiling, mitochondrial respiration assays, and transcriptomic indicators (e.g., redox‑sensitive gene signatures) could yield standardized biosignatures of PBMT efficacy. Establishing such objective readouts would facilitate precision dosing through real-time feedback systems.

From a regulatory standpoint, PBMT devices vary in wavelength calibration, safety certifications, and class categorizations across international jurisdictions. Disparities in approval criteria have led to unstandardized device outputs, influencing both safety outcomes and clinical performance [32]. Furthermore, the market proliferation of home-use or aesthetic devices without adequate thermal or irradiance controls poses safety risks and undermines clinical credibility. Consolidated clinical practice frameworks, guided by unified energy threshold recommendations, are essential to mitigate these variances. In multi-center clinical trials, integrating PBMT into standard care (e.g., oncology supportive therapy or neurorehabilitation) requires harmonization of operational parameters, training, and clinical endpoints. Current inconsistencies in endpoint definitions, for instance, between wound closure measurements and patient-reported outcomes, hinder pooled analyses and meta-analytic synthesis [89].

Despite these challenges, several innovations promise to advance PBM science toward precision and mechanistic sophistication. Multi-wavelength and pulsed-light systems now allow selective modulation of cytochrome and flavoprotein targets, expanding spectral adaptability [17]. The combination of PBMT with neurostimulation, regenerative scaffolds, or pharmacologic agents has shown synergistic metabolic and electrophysiological effects [77]. AI-based computational models are emerging as predictive frameworks for photon–tissue interactions, optimizing dosage predictions based on birefringence, hemoglobin saturation, and scattering coefficients.

In the neurocognitive domain, transcranial PBMT trials are transitioning from exploratory to mechanistic, correlating cortical hemodynamic shifts with specific mitochondrial and neuroplastic markers [7, 8]. Likewise, immune-modulated conditions such as rheumatoid arthritis, multiple sclerosis, and chemotherapy-induced mucositis continue to reveal subtype‑specific benefits through anti-inflammatory and cytokine-modulating mechanisms [88]. Expanding these findings into precision-guided clinical frameworks, grounded in dosing calibration, patient stratification, and multi-omics biomarker integration, represents the frontier for PBMT innovation. Ultimately, the pathway from empirical efficacy to mechanistic precision hinges on systematic parameter optimization, harmonized dosimetry standards, and biomarker-driven patient selection. Future studies must integrate molecular validation with clinical endpoints, transitioning PBMT from an empirical adjunct into a predictively controllable phototherapeutic modality.

## Conclusions

Photobiomodulation therapy has progressed from an empirical, supportive treatment to a scientifically grounded therapeutic platform with diverse biomedical implications. In dermatologic, musculoskeletal, neurological, ophthalmic, oncologic, and immunoregulatory fields, it has measurable effects on mitochondrial function, redox signaling, and tissue restoration. The breadth of translational evidence now supports PBMT as a biologically active approach that can modulate inflammation, enhance cellular recovery, and promote system-wide homeostasis. However, the heterogeneity of clinical outcomes underscores the importance of context, disease state, tissue type, and patient characteristics, all of which shape photobiological responsiveness. Persistent challenges include methodological inconsistency, lack of standardized dosimetry, and insufficient biomarker validation. The dose–response relationship remains non-linear, reflecting the hormetic nature of PBMT, where suboptimal dosing may yield null or inhibitory outcomes. Furthermore, mechanistic pathways, particularly those linking mitochondrial activation to immune regulation and neuroplasticity, require deeper elucidation through multi-omics and imaging-guided studies. Future translational progress will depend on rigorous, multicenter studies employing harmonized protocols and mechanistic endpoints. The integration of computational modeling, optical tissue diagnostics, and personalized irradiation strategies could transform PBMT from a generalized intervention into a precision-modulated biotherapy. Ultimately, the evolution of PBMT lies in bridging its molecular mechanisms with clinically reproducible outcomes, enabling rational, quantitatively optimized treatment frameworks that fulfill its potential as a safe, non-invasive, and mechanism-driven therapeutic modality across medical disciplines.

## Data Availability

The data presented in this study are available in the referenced articles.
